# Tumor-associated mesenchymal stem/stromal cells in tumor microenvironment and carcinogenesis

**DOI:** 10.1186/s40164-025-00688-7

**Published:** 2025-07-17

**Authors:** Li Sun, Xiaoli Cao, Baocheng Zhou, Jingyu Mei, Xinlan Zhao, Yuanyuan Li, Yongliang Yao, Mei Wang

**Affiliations:** 1 Department of Clinical Laboratory, Kunshan First People’s Hospital, Affiliated to Jiangsu University, Kunshan, 215300 China; 2 Department of Laboratory Medicine, Affiliated Tumor Hospital of Nantong University, Nantong, 226321 Jiangsu Province China; 3 Department of Medical Laboratory, Lianyungang Maternal and Child Health Hospital, Lianyungang, 222000 Jiangsu Province China; 4 Department of Laboratory Medicine, School of Medicine, Jiangsu University, 301 Xuefu Road, Zhenjiang, 212013 Jiangsu Province China

**Keywords:** Mesenchymal stem/stromal cells, Tumor microenvironment, Heterogeneity, Tumor initiation and progression, Clinical implications

## Abstract

Mesenchymal stem/stromal cells (MSCs) possess significant potential in regenerative medicine, attributed to their inherent capacity for site-specific homing to inflammatory regions, diverse differentiation abilities, and immunomodulatory functions. Tumors represent a substantial threat to human health, and therapeutic options remain limited. The inherent ability of MSCs to migrate towards tumor sites has been extensively utilized in cancer therapies. However, MSCs have shown ambiguous effects on tumors and contribute to the tumor microenvironment by trans-differentiation into different stromal cell types. Tumor-associated MSCs (TA-MSCs), derived from various tumor tissues, have been identified for their role in promoting tumor progression by interacting with tumor cells and other stromal components. As integral components of the tumor stroma, TA-MSCs provide a novel perspective for elucidating the mechanisms underlying malignancy. This review enhances our comprehension of TA-MSCs in solid tumors by summarizing evidence on their existence, differences from normal MSCs, heterogeneity, and roles in tumor initiation and progression. Furthermore, this review underscores the potential clinical implications of TA-MSCs for tumor diagnosis, prognosis prediction, and therapy.

## Background

Mesenchymal stem/stromal cells (MSCs) are well-known as a subpopulation of stromal cells with capacities of self-renewing and differentiating into multiple stromal cells. First isolated from bone marrow cell cultures by Friedenstein’s team in 1970, MSCs were termed “colony-forming unit fibroblasts (CFU-F)” or “stromal precursor cells” for their capacity to form fibroblast colonies and differentiate into osteoblast [1, 2]. Over time, extensive research has elucidated their multipotency, leading to the adoption of the term “mesenchymal stem cells” in the 1990s [3]. Although initially discovered in bone marrow, MSCs have since been found in almost all tissues [4]. In 2006, the International Society for Cellular Therapy (ISCT) set criteria for identifying isolated MSCs, which involve adherence to plastic in standard culture conditions, the ability to differentiate into osteoblasts, adipocytes, and chondroblasts, and expression of surface markers CD73, CD90, and CD105, while not expressing CD34, CD45, CD14, CD11b, CD79α, CD19, and HLA-DR [5]. The application of this set of criteria at least ensured consistency and comparability among MSCs from different tissues and laboratories. Recognizing the roles of MSCs in inflammation and immune regulation during tissue repair, the ISCT released guidelines in 2013 for their immunological characterization in clinical applications [6]. In contrast to earlier criteria, the guidelines emphasize the plasticity and heterogeneity of MSCs, shifting the focus away from their stemness property to their pivotal role as stromal cells in tissue regeneration by modulating inflammatory responses and the immune system [7].

In 1986, American surgeon Harold Dvorak introduced the idea that “tumors are wounds that never heal”. Drawing parallels between tumors and chronic wounds, Dvorak noted several similarities, including cell proliferation, angiogenesis, extracellular matrix (ECM) formation, leukocyte infiltration, and cytokine secretion [8]. As reviewed by Li *et al.*, MSCs participate in distinct stages of tumor “wound” progression [9], underscoring their integral role within the tumor microenvironment (TME). Remarkably, the administration of exogenous MSCs has been found to accelerate tumorigenesis in helicobacter pylori (*H. pylori*)-infected chronic gastritis mouse models [10]. In rat primary liver cancer models induced by diethylnitrosamine (DEN), a distinct population of AIF1^+^CSF1R^+^ MSCs was identified during the chronic inflammation stage. These MSCs cooperate with macrophages to establish an inflammatory niche that drove hepatocarcinogenesis [11], providing compelling evidence that endogenous MSCs in chronic inflammation actively stimulate tumorigenesis. In recent years, scholarly attention has grown regarding the characterization and extraction of MSCs from tumor tissues, known as tumor-associated MSCs (TA-MSCs). Although existing reviews address MSCs in tumor progression, this review specifically focuses on TA-MSCs, aiming to thoroughly elucidate their relationship with tumors. We will summarize the characteristics of TA-MSCs, their distinctions from normal MSCs, their inherent heterogeneity, and their significant roles in tumor initiation, progression, and metastasis, along with their potential clinical implications. Progress in this area will deepen our comprehension of TA-MSCs’ roles in tumorigenesis, aiding in identifying therapeutic targets and devising strategies to enhance clinical outcomes.

## TA-MSCs

Since Paget’s introduction of the “seed and soil” hypothesis in 1889 [12], stromal cells surrounding tumors are increasingly recognized as key players in tumor development, rather than mere bystanders. Naïve MSCs are distributed in almost all tissues, positioning MSCs as integral components of tumor stroma. Furthermore, tumorigenesis is essentially the outcome of chronic inflammation evolution [13]. Naïve MSCs exhibit selective migration to inflammatory sites, making it unsurprising that they are preferentially recruited into tumor sites and incorporated into tumor stroma. The term “TA-MSCs” is now used to describe resident MSC-like stromal cells within tumors. A growing body of research have successfully identified TA-MSCs, and elucidated their functions and regulatory mechanisms, highlighting their critical roles in tumorigenesis [14, 15].

### Evidences for the existence of TA-MSCs

The presence of TA-MSCs has been increasingly corroborated through diverse experimental methodologies, with direct isolation from clinical specimens serving as the gold standard. Initially identified in gastric cancer [16], TA-MSCs have subsequently been verified in a variety of solid tumors [17–19]. Moreover, their presence has been systematically demonstrated in metastatic contexts, such as lymph node and liver metastases in breast cancer [20], lymph node and ovarian metastases in gastric cancer [21, 22], and in metastatic body fluids, such as ascites in ovarian cancer [23–25]. Preclinical models have further substantiated these findings, including genetically engineered models where TA-MSCs have been isolated from transgenic mice with primary and metastatic tumors [26, 27], cell line-derived xenografts, such as those for glioma [28], prostate cancer [29, 30], and osteosarcoma [31], as well as in chemical compound-induced carcinogenesis mouse models [32]. This comprehensive body of evidence confirms TA-MSCs as pervasive stromal components within TMEs. Table 1 summarizes the isolation of TA-MSCs from clinical samples, while Table 2 provides details from tumor mouse models.

**Table 1:** Summary of TA-MSCs isolation from clinical samples

Tumor tissues	Isolation methods	Characterization	Function	Refs.
Primary head neck squamous cell cancer	Digesting tissues with collagenase IV to prepare single-cell suspensions for cell culture	CD90/CD29/CD105/CD73^+^; CD31/CD45/CD133^−^; exhibits adipogenic and osteogenic differentiation potential	Inhibiting the anti-tumor immune response through IDO-mediated suppression of T cells proliferation in vitro; promoting tumor growth in vivo	[33]
	Digesting tissues with collagenase type II followed by treatment with Ringer solution containing dispase to prepare cell suspension for culture	CD73/CD90/CD39^+^; exhibits trilineage differentiation	Inhibiting cell proliferation and IFNγ secretion of T cells potentially through adenosine in vitro	[34]
	Tissues were finely chopped (1–2 mm) and digested with collagenase II and dispase to generate a single-cell suspension for adherent culture	CD73/CD90/CD105/vimentin/S100A4^+^; CD14/CD19/CD34/CD45/HLA-DR^−^; trilineage differentiation	Elevated IL-6, IL-8, TNFα, SDF-1α, and IFNγ; minimal T-cell-modulating cytokines; promotes tumor growth in vivo	[35]
	Tissues were digested by dispase and then cut into 1 mm^3^-sized pieces for cell culture	CD29/CD73/CD90/CD105/STRO-1^+^; CD34/CD45^−^; trilineage differentiation potential	Promoting tumor metastasis by increased expression of CPNE7 and secretion of CXCL8 in vivo	[36]
Primary glioma	Tissues were trypsin-digested and filtered (70 μm) to prepare single-cell suspensions for culture	Immunofluorescence staining for CD44 and CD105	Increasing tumor cell expression of PD-L1 and angiogenesis in vitro	[37]
	Tumor samples were processed (minced/dissociated/filtered) to yield single-cell suspensions for culture	CD73/CD90/CD105^+^; CD133/CD31^−^; trilineage differentiation	Promoting CSC proliferation and self-renewal in vitro; facilitating CSC tumorigenicity and mesenchymal features in vivo	[38]
Primary endometrial cancer	Digesting tissues with type I collagenase followed by filtration through a 70 μm and 40 μm cell strainers to collect single cells for cell culture	CD44/CD73/CD90/ CD105/HLA-ABC^+^; CD34/CD45/HLA-DR^−^; trilineage differentiation	Exhibiting immunosuppressive effects by expression of PD-L1 and secretion of cytokines in vitro	[39]
Primary gastric cancer	Tissues were cut into 1-mm^3^ pieces for adherent culture	CD73/CD90/CD105/CD44/CD29^+^; CD34/CD45^−^; trilineage differentiation; normal karyotype; non-tumorigenic	NA	[16]
	Fibroblasts were isolated from 5 mm tissue pieces through enzymatic digestion followed by magnetic bead sorting	CD73/CD105/CD90/CD44/CD29^+^; CD45/CD34^−^; trilineage potential	Specifically supporting tumor cell growth and tumorigenicity in vitro and in vivo	[40]
	1-mm^3^ tissues for adherent culture	CD29/CD44/CD90/ CD105^+^; CD14/CD34/CD45^−^; osteo/adipogenic potential	Secreting IL-6/IL-8 to drive M2 polarization to promote cancer cell EMT in vitro	[41]
	1-mm^3^ tissues for adherent culture	CD29/CD44/CD90/CD105^+^; CD14/CD34/CD45^−^; osteo/adipogenic potential	Promoting cancer cell proliferation, migration and angiogenesis in vitro	[42]
	1-mm^3^ tissues for adherent culture	NA	Mediating 5-FU resistance through CTCF-PD-L1 in vitro/vivo	[43]
	1-mm^3^ tissues for adherent culture	NA	Promoting enrichment of CSC of gastric cancer through PD-L1-CTCF in vitro and in vivo	[44]
Primary lung cancer	Minced tissues for adherent culture	CD73/CD90/CD166^+^; CD14/CD19/CD34/CD45/HLA-DR^−^; trilineage differentiation	Promoting tumor cell invasion, migration in vitro as well as tumorigenesis and metastasis in vivo	[45]
	Tissues were digested with collagenase II/IV and DNase I, filtered (100 μm), and adherent cells were cultured long-term	CD90/CD105/CD73^+^; CD14/CD20/CD34/CD45^−^; trilineage differentiation	Contributing to chronic inflammation in the TME in vitro	[46]
	Digesting tissues with collagenase II and IV and DNase I to prepare single-cell suspensions for cell culture	CD105/CD73/CD90/ vimentin/α-SMA^+^; Lin^−^; trilineage differentiation	Promoting CD56^dim^ phenotype with altered degranulation/receptor expression, while suppressing IFNγ in CD56^bright^ NK cells in vitro	[47]
	Tissues were minced into 1 mm³ pieces and digested for adherent culture	Vimentin^+^/Keratin^−^; adipo/osteogenic potential; CD90/CD105/CD44^+^; CD45/CD19/CD14/CD34^−^	Promoting cell viability, proliferation, and migration as well as inhibiting cell death in vitro	[48, 49]
	Tissues were digested with collagenase II/IV + DNase, filtered (100 μm), and adherent cells cultured for MSC expansion	CD90^ +^ CD166^ +^ CD105^+^ CD44 ^+^ CD73 ^+^ with trilineage potential; vimentin + α-SMA−; normal karyotype and non-tumorigenic	Enhancing tumor metastasis in vivo	[50]
Primary intrahepatic cholangiocarcinoma	Minced tissues were digested (collagenase IV/DNase), filtered (100 μm), and SSEA-4^ +^ cells isolated by FACS	Positive for SSEA-4	Supporting survival of chemotherapeutic cholangiocarcinoma cells and inhibiting their apoptosis through AMPK/mTOR‑dependent autophagy in vitro	[51]
Primary breast cancer	1-mm^3^ tissues for adherent culture	CD73/CD44/CD29/CD105/CD90^+^; CD45/CD11b/CD34/CD133/CD31/HLA-DR^−^; adipo/osteogenic potential	Elevating PGE2/TGF-β/IL-10 in peripheral blood lymphocytes (PBLs) while enhancing their proliferation in vitro	[52]
	Using human CD45 and CD271 microbead kits to sort CD45^−^CD271^+^ MSCs	Positive for CD271, but negative for CD45	Polarizing undifferentiated myeloid-monocytic cells into M2-type macrophages to potentiate tumor grow in vitro and in vivo	[53]
	Collagenase I-digested single cells for culture	Fibroblastic morphology; positive for CD90/CD29/CD105/CD73/CD166, α-SMA/vimentin/ECM proteins; negative for CD31/CD144/CD14/CD45/HLA-DR, epithelial markers; trilineage differentiation	Enhancing tumorigenesis and mammosphere formation in vitro	[18]
	Tissues were cut into 1 mm^3^-sized pieces for adherent cell culture by replacing the medium every three days after the initial plating	Spindle shape; CD13/CD29/CD44/CD105/HLA-I^+^; CD4/CD10/CD14/CD31/CD34/CD38/HLA^−^DR-; osteogenic/adipogenic potential	Enhancing tumor cell proliferation and migration in vitro	[54]
	Minced tissues were collagenase I-digested for adherent culture	Spindle shape; CD44/CD105/CD166^+^; CD14/CD34/CD45^−^; chondrogenic/hepatogenic potential	Increasing the percentage of CD4^+^CD25^high^ Foxp3^+^ Treg in vitro	[55]
Primary colorectal cancer	Collagenase IV/DNase I-digested tissues filtered (100 μm) for single-cell culture	CD73/CD90/CD105/ CD29^+^; CD45/CD31/EpCAM^−^; FSP1/α-SMA/vimentin^+^; trilineage differentiation	Promoting tumor cell proliferation and metastasis in vitro and in vivo	[56]
	Minced tissues were collagenase IV-digested and 70 μm-filtered for single-cell culture	CD73/CD90/CD105/ CD44^+^; CD45^−^; trilineage differentiation	Helping cancer cells defend against senescence and facilitating tumor growth in vitro and in vivo	[57]
	Tissue sections were collagenase IV-digested, 70 μm-filtered, and cultured as single cells	Spindle-shaped; CD105/CD90/CD73/ CD44^+^; CD45^−^; tri-lineage differentiation potentials	Enhancing motility/EMT/stemness/angiogenesis in vitro and growth/metastasis in vivo via IL-6/JAK2/STAT3	[19]
Primary colon cancer	Tissues were digested with collagenase and filtered to obtain cell pellets for culture	Fibroblast-like morphology; CD166/ CD13/CD44/CD90/CD73/CD14/Oct4/Nanog/ Bmi1^+^; CD31/CD45/ CD34/CD133^−^; osteogenic/adipogenic differentiation	Promoting migration and invasion in vitro and Stimulating growth and metastasis in vivo through IL-6-Notch-1-CD44 pathway	[58]
Primary ovarian cancer	Plastic adherence selection	CD105/CD90/CD73^+^; CD45/CD34/CD14/ CD19^−^; trilineage differentiation	Increasing migration, invasion and survival of tumor cells in vitro as well promoting cancer metastasis in vivo	[59]
	Minced tissues were collagenase IV-digested, 70 μm-filtered, and cultured adherently	CD73/CD90/CD105/CD59/CD13/CD49b^+^; CD34/CD45^−^; multilineage differentiation potential; normal karyotype; non-tumorigenic	NA	[60]
	Tissues were mechanically dissected and filtered to prepare single cell suspension for adherent cell culture	CFU-fibroblastic morphology; CD105(SH2)/CD73/ CD90/CD44^+^; CD14/CD45/CD34/ CD133^−^; trilineage differentiation	Enhancing tumor growth and tumorigenicity in vivo	[61]
	Plastic adhesion cell culture and then sorted by FACS using the MSC phenotyping kit	CD73/CD90/CD105^+^; CD14/CD20/CD34/ CD45^−^; impaired differentiation	Inducing chemoresistance and polarizing macrophages towards M2 type to potentiate tumor progression in vitro and in vivo	[62]
Primary cervical cancer	Tissues were dissected, trypsin-EDTA digested, 100-µm filtered, and centrifuged for cell culture	CD29/CD44/CD49b/CD58/CD166/HLA-ABC^+^; CD31/CD45/CD133/CD62L/HLA-DR^−^; trilineage differentiation	Inhibiting HLA-I expression and protecting tumor cells from cytotoxic T cell attacking in vitro	[63]
	1-mm^3^ tissues for adherent culture	Spindle shape; CD13/CD29/CD44/CD105/HLA-I^+^; CD10/CD14/CD31/CD34/CD38/HLA-DR^−^; differentiation into osteocytes, adipocytes and hepatocytes	NA	[64]
Primary liver cancer	Minced tissues were collagenase I-digested for culture, with nonadherent cells removed at 24–48 h	Fibroblastic morphology; D29/CD73/CD90/CD105/CD166^+^; CD14/CD31/CD45/ CD144^−^; adipogenic and osteogenic differentiation	Enhancing cancer stemness and tumorigenesis through lncRNA-MUF in vitro and in vivo	[65]
	Minced tissues were collagenase I-digested for culture, with nonadherent cells removed at 24–48 h	Fibroblastic morphology; D29/CD73/CD90/CD105/CD166^+^; CD14/CD31/CD45/ CD144^−^; adipogenic and osteogenic differentiation	Promoting tumor cell proliferation, tumor sphere formation, migration, invasion and metastasis though SA1004-miR-155-SOCS1-MMP9 in vitro and in vivo	[66]
	Minced tissues were collagenase I-digested for culture, with nonadherent cells removed at 24–48 h	Positive for CD29, CD73, CD90, and CD105	Accelerating HCC growth and metastasis through a DNM3OS/KDM6B/TIAM1 axis in vitro and in vivo	[67]
Primary ameloblastoma	Tissue fragments were digested (collagenase/dispase), 70-µm filtered, and centrifuged for single-cell culture	Spindle; CD29/CD44/CD73/CD90/STRO-1/CD105/CD146/ SSEA4^+^; CD34^−^; positively staining for Vimentin, LIN28, SOX2, CD44, CD29, SSEA4, STRO-1, and ALDH1	Promoting tumor cell stemness through secreting IL-6-eliciting STAT3 and ERK1/2 to induce EMT in vitro and in vivo	[68]
Primary neuroblastoma	Mechanical dissociation and collagenase type II treatment to prepare single cell suspension for adherent cell culture	Spindle and proliferative capacity; osteogenic/chondrogenic differentiation; CD73/ CD90/CD105/HLA-I^+^; a CD34/CD45/CD14/CD31/ HLA-DR^−^	More suppressive on the activated T cells in vitro	[69]
	Tissues were mechanically dissociated and digested by collagenase type II to prepare single cell suspension for adherent cell culture	Positive for CD90, CD73, CD29, CD146 and CD105; expressing stemness markers Nanog, Sox2 and Oct4	Expressing the checkpoint ligands PD-L1 and PD-L2, macrophage checkpoint CD47, the mesenchymal immunomodulatory transcriptional coactivator WWTR1 and MHC-1; inhibiting the cytolytic activity of NK cells in vitro	[70]
Primary esophageal carcinoma	1–3 mm³ tissue sections were collagenase IV-digested for adherent culture	Long and spindle-shaped; CD13/CD29/CD44/CD105+; CD34/CD45/CD133 /HLA-DR-	NA	[71]
Primary prostate cancer	Minced tissues were enzymatically digested, 70 μm-filtered, and expanded in culture	CD73/CD90/CD105+; CD14/CD20/CD34/CD45/HLA-DR-;	NA	[72]
Primitive neuroectodermal tumor	Tissues were mechanically dissociated into single cells for adherent cell culture	Spindle shape; positive for CD105, CD90, and CD73, and negative for CD45, CD31 and NG2; tri-lineage differentiation potency	NA	[73]
Primary ewing sarcoma	Bone marrow mononuclear cells were Ficoll-isolated for adherent culture	Plastic adherence; CD105/CD90/CD73/CD166^+^; CD34/CD45/CD19 /HLA-DR^−^; trilineage differentiation	NA	[74]
Primary osteosarcoma	Minced tissues were collagenase/dispase-digested, 100-µm filtered for single-cell culture	CD105/CD73/CD90/CD44/CD166/HLA-I^+^; CD45/CD34/CD14/CD19/HLA-DR/CD31^−^; trilineage differentiation; FSP/α-SMA/vimentin+; normal karyotype	NA	[17]
Primary atypical teratoid/rhabdoid tumor	Tissue was cut into 1 mm^3^ for adherent cell culture	Fibroblast-like cells; CD29/CD44/CD90/CD105/CD166+; CD31/CD34/CD45-	Facilitating tumor cell migration through exosomal miR-155 eliciting SMARCA4 Pathway in vitro	[75]
Primary pituitary adenomas	Tissues were disaggregated and accutase-treated for culture	Fibroblast-like morphology; Not expressing pituitary parenchymal progenitor markers; CD90/CD105/CD44^+^; CD34/CD45/HLA-DR^−^; expressing vimentin/SOX2/POU5F1; osteogenic/adipogenic differentiation	NA	[76]
Metastatic lymph nodes of gastric cancer	1-mm^3^ tissues for adherent culture	CD44/CD29/CD105^+^; CD34/CD45/CD14-; adipogenic/osteogenic differentiation	Promoting migration, invasion and lymphangiogenesis in vitro	[21]
Ovarian metastasis of gastric cancer	1-mm^3^ tissues for adherent culture	CD73/CD90/CD105^+^; CD34/CD45/HLA-DR^−^; expressing stem cell-associated genes and mesenchymal lineage-associated genes; increased cumulative population doubling level; without tumorigenesis; trilineage differentiation	NA	[22]
Lymph node metastasis and liver metastasis of breast cancer	Minced tissues were collagenase-hyaluronidase dissociated, RBC-lysed, trypsin-EDTA/DNase1-treated, and 40-µm filtered for culture	Spindle-shape; trilineage differentiation potentials; normal karyotype; positive for CD44, CD90, CD73, and CD105	Enhancing tumor growth and metastasis depending on DDR2 in vitro and in vivo	[20]
Ascites fluid of ovarian cancer	Ascites was centrifuged and plated for one week followed by EpCAM^ −^ cells sorted and passaged	Spindle-shaped; CD45^−^; CD73/CD105/ CD29/CD90^+^	Promoting tumor cell chemotherapy resistance through secreting CCL2/5 to trigger IL-6/PYK2 dependent mechanisms in vitro and in vivo	[25]

**Table 2:** Summary of TA-MSCs isolation from tumor mouse models

Tumor tissues		Isolation methods	Characterization	Function	Refs.
Primary breast cancer		FACS sorting	CD45^−^CD11b^−^CD44^+^CD106^+^Sca1^+^	Promoting differentiation of M-MDSCs into highly immunosuppressive M2-type macrophages to promote tumor growth in vitro and in vivo	[53]
Primary breast cancer and metastatic lung	Digestion with collagenase II and hyaluronidase; cell suspension culture; purification by immunomagnetic separation for adherent CD45^−^ cells		Spindle-like morphology; CD29^+^CD44^+^CD140a^low^Nestin^+^Sca1^low^Lineage^−^CD11b^−^CD31^−^CD34^−^CD45^−^phenotype; differentiating into adipocytes and osteoblasts	Promoting tumor lung metastasis through recruitment of neutrophils and formation of NETs through release of C3 in vivo	[26]
Primary glioma	FACS sorting		Fibroblast-like shape; Lin-Sca-1^+^CD9^+^CD44^+^CD166^+/−^; tri-lineage differentiation	Increasing tumor cell proliferation in vitro and in vivo	[28]
	Tissue dissection for cell culture and remove nonadherent cells after 24 h		Sca-1^+^CD9^+^CD45^−^CD11b^−^CD31^−^, and NG2^−^; mesenchymal differentiation potency	Providing the mesenchymal elements of the vascular niche *ex vivo*	[77]
Primary pancreatic cancer	Tissues were minced and digested with collagenase, and then sorted by FACS		Positive for CD73, CD90, CD49a and CD44, negative for CD45^−^; adipogenic and osteogenic differentiation capacities	Promoting infiltration of monocytes/macrophages and differentiation to enhance tumor growth in vitro and in vivo	[26]
	Isolated from primary tumor-derived CAFs by co-expression of CD90, CD49α, CD44, and CD73		Capable to form colonies; multipotent differentiation	Promoting tumor progression in vitro (proliferation/invasion/migration) and in vivo (growth/metastasis)	[78]
Primary prostate cancer	Tumors were minced and digested with collagenase for subsequent cell culture		Fibroblastoid, plastic-adherent CD45^ −^ CD81^ +^ Sca-1 ^+^ cells with proliferative and adipo/osteogenic potential	Contributing to tumor-associated stroma formation *ex vivo*	[29]
	Tissue pieces were 200-mesh filtered, then CD105 ^+^ cells magnetically sorted for culture		CD44/CD73/CD90/CD105^+^; CD14/CD34/CD45/MHC-II^−^; trilineage differentiation	NA	[79]
	Tissue cubes were 200-mesh filtered, then CD105^ +^ MSCs magnetically sorted		Highly expressing SDF-1, CXCR4 and VEGF	NA	[30]
Primary osteosarcoma	3 mm³ tissue pieces were 200-mesh filtered, centrifuged, and CD105 + cells magnetically sorted for culture		CD34/CD45/CD14/MHC-II^−^; CD44/CD73/CD105/CD90^+^; trilineage differentiation	NA	[31]
Primary oral mucous carcinogenesis	Minced tissues were collagenase IV-digested, 70 μm-filtered, and cultured as single cells		CD90/CD29+; CD45/CD34−; adipogenic/ osteogenic differentiation	Inhibiting T-cell proliferation and enhancing tumor cell proliferation in vivo and in vitro	[32]

**Fig. 1:**
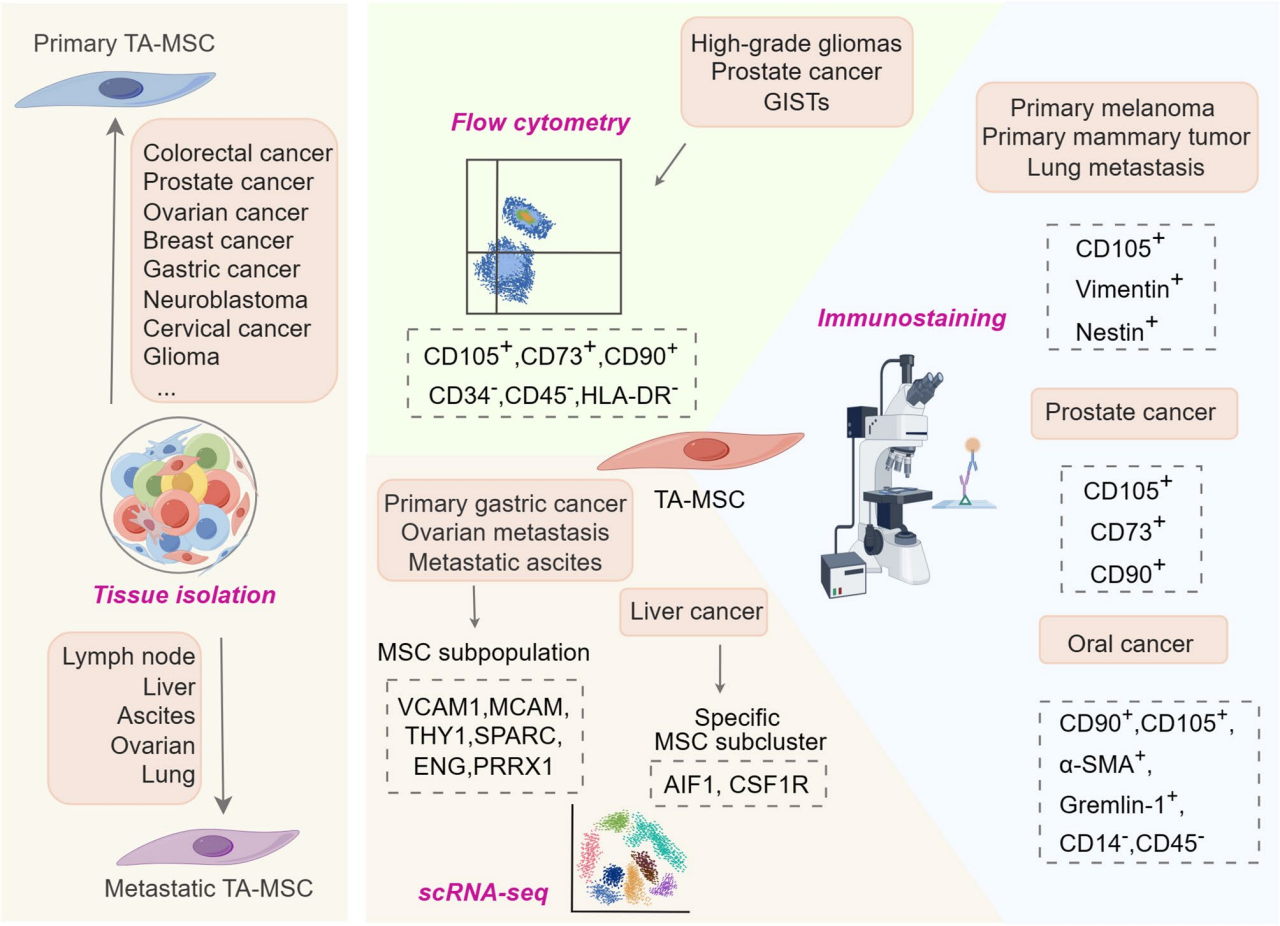
Evidence supporting the existence of TA-MSCs. Direct evidence stems from isolation of MSC-like cells from various primary tumors and metastatic sites. Indirect evidence is obtained through flow cytometry, immunostaining, and scRNA-seq analysis of MSCs in primary tumor tissues and metastases

Besides directly isolating TA-MSCs, many studies have shown their existence indirectly through techniques like immunostaining and flow cytometric analysis. Immunostaining for MSC markers (CD90, CD105, and gremlin-1), myofibroblast marker (α-SMA) and hematopoietic lineage markers (CD14 and CD45) confirms MSC presence in primary oral cavity and oral pharyngeal squamous cell carcinoma [80]. Immunofluorescence assays identify CD105^+^Vimentin^+^ MSCs in lung metastases of a mouse melanoma model [81] and Nestin^+^ MSCs in metastatic lung tissues of MMTV-PyMT transgenic mouse mammary tumors [27]. Multiplex immunofluorescence assays for MSC markers CD73, CD90, and CD105 confirm the presence of TA-MSCs in primary prostate cancer tissues [82]. Flow cytometry, utilizing MSC markers CD105, CD73, and CD90 combined with negative expression of hematopoietic lineage markers, has been employed to detect and quantify TA-MSCs in high-grade gliomas [83], primary prostate cancer [72], and gastrointestinal stromal tumor [84]. Recently, single-cell RNA sequencing (scRNA-seq) has become a powerful approach to analyze tumor tissues at single-cell level [85]. A subpopulation of MSCs expressing specific marker genes such as THY1, SPARC, ENG, PRRX1, VCAM1, and MCAM has been identified in primary lesions, ovarian metastases, and peritoneal metastases of gastric cancer [86]. A separate study isolated CD90^+^ MSCs from both primary breast cancer and normal tissues, using scRNA-seq to identify MSCs subclusters linked to cell matrix adhesion in tumor sites [87]. In a rat chemical-induced primary liver cancer model, scRNA-seq technology identified a distinct group of endogenous MSCs contributing to inflammatory environment mediated occurrence of liver cancer [11]. Visualization of MSCs in mouse tumor models has been accomplished using XYZeq, an innovative technique that incorporates spatial metadata into scRNA-seq libraries to ascertain the spatial positioning of individual cells within tumors [88]. Collectively, both direct isolation and indirect detection studies provide compelling evidence confirming the presence of TA-MSCs (Fig. 1).

### Differences between TA-MSCs and their normal counterparts

Given that TA-MSCs have been established as integral components of the TME, researchers are eager to delineate the biological disparities between TA-MSCs and their normal counterparts in terms of their behavior, molecular expression profiles, and functions (Table 3).

**Table 3:** Differences between TA-MSCs and various MSC controls in terms of biological behavior, molecular expression profile, and function

Controls	TA-MSCs	Biological behavior	Molecular expression	Functions	Refs.
BM-MSCs	Osteosarcoma	Increasing stemness properties; reducing adipogenic differentiation capacity	NA	NA	[89]
	Head neck squamous cell cancer	Similar	Higher proportions of CD90^+^ cells	Comparable in chemoattraction and suppression of T cells proliferation in vitro	[33]
		Similar	NA	Enhanced capacity to promote tumor growth in vivo	[35]
	Gastric cancer	Similar	Higher YAP expression	Potentiating cancer cell migration, invasion and HLEC tubule formation in vitro	[90]
		Increased number of cell organelles; highly proliferative activity	Higher levels of PCNA expression	NA	[16]
	Prostate cancer	Smaller in size at the earliest passages; less efficiency in adipogenesis under normoxia	NA	NA	[72]
		NA	Similar in upregulating PD-L1 and PD-L2 on cell surface in response to IFNγ and TNFα	Comparable in suppressing T cell proliferation in vitro	[82]
	Lung cancer	NA	NA	Stimulating cancer cell proliferation, viability and migration in vitro	[49]
		Similar	NA	Greater capacity to promote metastasis in vivo	[50]
	Ovarian cancer	Losing their multipotency	Increased secretion of CXCL1, CXCL2, and IL-8	Promoting chemoresistance and impairing anti-tumor immunity by polarization of M2 type macrophages in vitro and in vivo	[62]
	Esophageal carcinoma	Growing faster	Increased expression of VEGF, Bmi-1, Nanog, Oct-4 and CK18	NA	[71]
	Neuroblastoma	Losing adipogenic differentiation capacity; similar in reaching senescence at an earlier phase	Similar in expression of stemness markers and oligodendrocyte marker but highly expressing EMT-, stemness- and Wnt pathway- related genes	More suppressive on the activated T cells in vitro	[69]
	Cervical cancer	Less efficiency in adipogenesis	Increased positivity for CD49b and CD54; higher secretion of IL-10	Comparable in suppressing HLA-I expression and capacity to protect tumor cells from cytotoxic T cell killing in vitro	[63]
		NA	NA	Similar in inducing tumor cell expression of CD73 in vitro	[91]
BM-MSCs and Wharton’s Jelly MSCs	Endometrial cancer	Similar	Highly expressing PD-L1 and PD-L2	Suppressing PBMC proliferation and the cytotoxicity of cytokine-induced killer cells in vitro	[39]
AD-MSCs	Breast cancer	Higher proliferation potential	Secreting a higher level of TGF-β, PGE2, VEGF and IDO, but lower levels of MMP2 and MMP9	Increasing peripheral blood lymphocyte proliferation, secretion of IL-10, TGF-β, and PGE2, and Treg cell percentage in vitro	[52]
	Ovarian cancer	Enhanced single-cell cloning and adipogenic potential	Increased ALDH^+^ cell percentage; elevated BMP2, BMP4, and BMP6 expression	Enhanced capacity to promote tumor growth and stemness in vitro and in vivo	[61]
UC-MSCs	Intrahepatic cholangiocarcinoma	NA	Overexpression of Herpesvirus entry mediator	Supporting cell survival of chemo-therapeutic cholangiocarcinoma cells and inhibiting their apoptosis in vitro	[51]
Local normal MSCs	Oral squamous cell carcinoma	NA	MMP1 enriched in TA-MSCs exosomes	Enhancing HUVEC angiogenesis in vitro and in vivo	[92]
	Ovarian Cancer	NA	Enhanced hypermethylation with chromatin/MET dysregulation	Promoting metastasis in vivo	[59]
		Shorten doubling time	Reduced expression of CD29, CD49a, and CD49d; increased expression of CD146/MCAM; differing in biological functions and transcriptional regulators; alteration in EGF-dependent morphological changes and KRT7 levels	NA	[60]
	Pancreatic cancer	Similar	Higher expression and secretion of IL6, Cox-2, TGFβ, and IL-10	Enhanced capacity to promote tumor growth through recruiting and polarizing macrophages in vitro and in vivo	[26]
	Breast cancer	Similar	Increased expression of IL-10 and TGF-β1;	Inducing Treg cell expansion; Increasing levels of IL-4, IL-10, TGF-β1, CCR4, CD25 and CTLA-4 in peripheral blood lymphocytes in vitro	[55]
	Cervical cancer	Similar	Higher secretion of IL-10	Enhanced capacity to downregulate HLA-I and promote tumor cell evasion from cytotoxic T cell attacking in vitro	[63]
	Primary ewing sarcoma	Similar	NA	NA	[74]
Paired NTA-MSCs	Colorectal cancer	Similar	Increased secretion of CCL7 and CCL8	Enhancing tumor cell expression of CCL5, and promoting tumor cell proliferation and metastasis in vitro and in vivo	[56]
	Head and neck squamous cell cancer	Similar	Reduced expression of CD39 and CD73 as well as production of immunosuppressive adenosine	Weakly inhibiting production of IFN-γ by T-cells in vitro	[34]
	Gastric cancer	Similar	Increased expression of pro-angiogenesis factors including VEGF, MIP-2, TGF-β1, IL-6, and IL-8	Enhancing cancer cell proliferation, migration and angiogenesis in vitro	[42]
		Similar	Differential expression of miRNAs	Enhanced capacity to promote tumor growth and migration in vitro and in vivo	[93]
	Lung cancer	Similar	Increased percentage of CD105 expression and highly secreting TGF-β	Enhanced capacity to promote tumor cell EMT, invasion in vitro and metastasis in vivo	[45]
		Similar	Different gene expression profiles	Comparable in promoting metastasis in vivo	[50]
	Squamous cell lung carcinoma	Similar	Similar	More immunosuppressive; reducing NK cell function and modulating NK phenotype in vitro	[47]
	Liver cancer	Similar	Differential expression of 2,121 genes	Promoting tumor growth and metastasis in vitro and in vivo	[66]
	Esophageal carcinoma	Increased growth activity	Higher expression of VEGF, Bmi-1, Nanog, Oct-4 and CK18	NA	[71]
	Cervical cancer	Similar	Expressing higher levels of CD39 and CD73	Strongly suppressing cytotoxic T-cells proliferation, activation and effector functions in vitro	[94]
		NA	NA	Induces TGF-β1/IL-10 in cervical cancer cells, thus evading T cell cytotoxicity in vitro	[95]

TA-MSCs and BM-MSCs have been directly compared in several tumors such as gastric cancer [96, 97], glioma [38, 98] and pancreatic cancer [26]. Although the majority of TA-MSCs exhibit similar morphologies, surface markers, and multilineage differentiation potentials as BM-MSCs, certain TA-MSCs demonstrate increased stemness characteristics, such as sphere formation capacity and tri-lineage differentiation abilities [17, 69, 72, 89]. While limited research has directly compared the biological behaviors of TA-MSCs and BM-MSCs, evidence indicates that TA-MSCs derived from prostate cancer and gastric cancer exhibit enhanced proliferative and migratory activities [79, 99]. Functionally, TA-MSCs exert oncogenic roles through various mechanisms, encompassing promotion of tumor growth, metastasis, tumor cell stemness, chemotherapy resistance, and immunosuppression [15]. However, the tumor-promoting capacity of TA-MSCs varies when compared to BM-MSCs. Research shows that TA-MSCs may have enhanced tumor-promoting effects than BM-MSCs in gastric cancer [42, 96], lung cancer [50], ovarian carcinoma [61] and neuroblastoma [100]. However, in breast cancer [101], prostate cancer [82], glioma [38], and head and neck squamous cell carcinoma (HNSCC) [33, 35], their effects appear similar. To elucidate the potential molecular mechanisms of TA-MSCs, expression profiles of genes, non-coding RNAs, and secreted factors have been compared between TA-MSCs and BM-MSCs [30, 42, 96, 101, 102]. Deregulated genes and circular RNAs (circRNAs) in gastric cancer-derived TA-MSCs are significantly linked to tumor progression. TA-MSCs release tumor-promoting inflammatory cytokines and chemokines, including IL-6, IL-8, VEGF, and SDF-1, enhancing their oncogenic characteristics [30, 42, 96, 101]. Although TA-MSCs and BM-MSCs exhibit similar pro-tumor effects in certain malignancies [38], employing BM-MSCs as a control may be inappropriate in these contexts. Nonetheless, a joint analysis of BM-MSCs and TA-MSCs can facilitate the identification of shared potential tumor-promoting factors.

Two others naïve MSC types, namely Adipose tissue-derived MSCs (AD-MSCs) and local tissue MSCs, are frequently used as controls to investigate TA-MSCs, particularly in studies investigating ovarian tumors and breast cancer [55, 60, 61, 103]. Breast cancer-TA-MSCs show higher levels of IL-10 and TGF-β compared to AD-MSCs, and they more effectively boost anti-inflammatory cytokine expression and Treg percentage in peripheral blood lymphocytes, indicating an increased potential to promote tumor growth [55]. RNA sequencing of ovarian carcinoma-TA-MSCs identifies a distinct predictive algorithm comprising six genes: Annexin A8-like protein 2 (ANXA8L2), Collagen Type XV Alpha 1 Chain (COL15A1), Cytokine Receptor Like Factor 1 (CRLF1), GATA Binding Protein 4 (GATA4), Iroquois Homeobox 2 (IRX2), and TGF‐β2. This algorithm holds promise for accurately distinguishing TA-MSCs from omental normal MSCs [103]. In research examining TA-MSCs from ovarian carcinoma and high-grade serous ovarian cancers (HG-SOCs), AD-MSCs, BM-MSCs, and MSCs from normal ovary or heart tissue were used as controls [60, 61]. TA-MSCs associated with ovarian carcinoma exhibit decreased CD90 and CD105 expression, alongside elevated stemness, improved single-cell colony formation ability, and heightened adipogenic differentiation potential [61]. TA-MSCs significantly enhance tumor growth in vivo and increase cancer cell stemness in vitro. PCR-based array analysis and validation assays show that TA-MSCs, unlike AD-MSCs, exhibit significantly elevated expression of bone morphogenetic protein (BMP) family proteins, which are key regulators in the oncogenic roles of TA-MSCs in tumor growth [61]. HG-SOCs-TA-MSCs display distinctive functional and molecular characteristics when compared to the control MSCs. Although they retain core MSC transcriptional regulators, these cells exhibit unique activation patterns, especially in genes associated with cancer. Notably, they demonstrate accelerated proliferation, increased differentiation into endothelial cell-like phenotypes, reduced expression of fibronectin-binding integrins, and elevated levels of CD146/MCAM [60]. These features may be associated with their roles in supporting tumor progression.

While naïve MSCs are commonly implicated in tumor progression, their utility as controls is limited in accurately representing physiological conditions. To more precisely capture the characteristics of TA-MSCs, phenotypic and functional comparisons are preferably conducted between TA-MSCs and MSCs derived from paired adjacent non-tumor tissues (NTA-MSCs). Compared to NTA-MSCs, TA-MSCs from gastric cancer [42], lung cancer [45, 47, 50], pancreatic cancer [26] and hepatocellular carcinoma (HCC) [66] exhibit a notable elevation in cytokines like IL-6, IL-8 and TGF-β, along with genes including MMP1, S100A4, GREM1, and LOXL2, all of which are associated with tumorigenesis. Additionally, a substantial number of microRNAs (miRNAs) are deregulated in TA-MSCs compared to NTA-MSCs in gastric cancer, with elevated levels of miR-221 identified as a critical mediator of their oncogenic activity [93]. Functionally, TA-MSCs from gastric cancer [93] and esophageal carcinoma [71] more effectively promote tumor cell proliferation, migration, and angiogenesis in vitro, as well as tumor growth and metastasis in vivo while TA-MSCs from pancreatic cancer uniquely induce alternatively activated macrophage polarization by promoting Arg1 expression [26]. However, in the context of lung cancer, both TA-MSCs and NTA-MSCs similarly influence cancer cell migration, gene expression, and primary tumor growth. Notably, TA-MSCs distinctly enhance cancer cell invasion, epithelial-to-mesenchymal transition (EMT), and stemness in vitro through indirect cell-cell interactions, and promote lymph node metastasis in vivo [45]. Conversely, the immunosuppressive effect of TA-MSCs on natural killer (NK) cells is more pronounced than that of NTA-MSCs in direct cell-cell contact systems, but this effect is reversed in indirect contact scenarios, potentially due to the suppression of NK cell receptors upon interaction with TA-MSCs [47]. In cervical cancer, TA-MSCs exhibit heightened immunosuppressive effects on CD8 ^ +^ T cell antitumor activity, which is attributed to increased expression of CD73 and CD39 [94], as well as a greater ability to inhibit the expression of HLA class I molecules (HLA-I) on the tumor cell surface [63]. This is in contrast to observations in head and neck squamous cell carcinoma (HNSCC), where TA-MSCs display reduced levels of CD39 and CD73 compared to NTA-MSCs [34]. Nevertheless, both TA-MSCs and NTA-MSCs similarly enhance CD73 expression in tumor cells [91]. In non-small cell lung carcinomas, it is noteworthy that not all patient-derived TA-MSCs exhibit an enhanced tumor-promoting function compared to their corresponding NTA-MSCs, potentially influenced by the properties of the tumors [50]. Overall, TA-MSCs generally exhibit a more pronounced tumor-promoting capacity compared to their paired NTA-MSCs. However, their functional impact can occasionally differ, ranging from comparable to diminished efficacy. This variability is likely influenced by factors such as the tissue source of the MSCs, the mode of culture system employed (whether direct or indirect cell-cell contact), and the specific tumor context.

## Heterogeneity of TA-MSCS

**Fig. 2:**
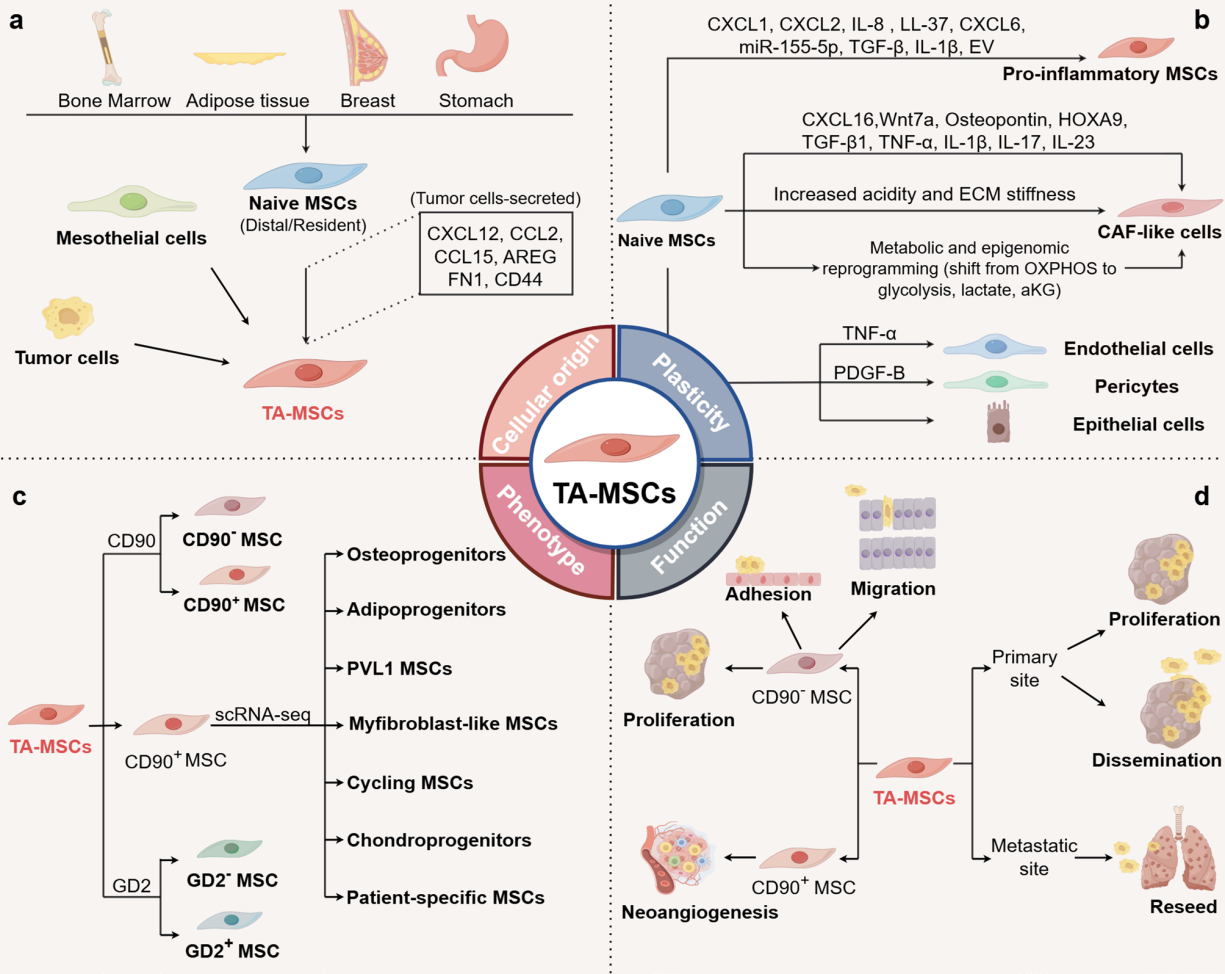
Heterogeneity of TA-MSCs. TA-MSCs exhibit heterogeneity in four distinct aspects: a, Cellular origin: Naïve MSCs, encompassing distal MSCs and resident MSCs, mesothelial cells and tumor cells are currently demonstrated to serve as cellular source of TA-MSCs; b, Plasticity: Upon engraftment into tumors, naïve MSCs undergo a stable transition into pro-inflammatory TA-MSCs and CAF-like cells through various mechanisms. Additionally, MSCs exhibit potential trans-differentiation into endothelial cells, pericytes, and epithelial cells within specific microenvironments. c, Phenotype: The phenotypical heterogeneity of TA-MSCs is evidenced by the surface markers like CD90 and GD2 as well as distinct MSC subclusters revealed by scRNA-seq analysis; d, Function: The functional heterogeneity in TA-MSCs arises from their diverse phenotypes and different sites

Heterogeneity of TA-MSCs is an important influencing factor contributing to intra-tumoral heterogeneity. TA-MSCs are remarkably heterogeneous in cellular origin, phenotype, plasticity, and function (Fig. 2).

### Cellular origin

Due to tropism towards inflammatory sites, naïve MSCs are naturally investigated as important sources of TA-MSCs. To assess whether naïve MSCs are potential cell origins of TA-MSCs, the first thing is to determine whether naïve MSCs effectively home to the tumor site. Evidences from systemic administration of luciferase or fluorescent protein, or fluorescent dye-labeled BM-MSCs or AD-MSCs into xenograft tumor models show selective engraftment of labeled MSCs in tumor sites, including both primary and metastatic sites [104–109]. This notion is further supported by the findings of increased chemotaxis of naïve MSCs towards tumor cells in vitro [106, 109]. More importantly, endogenous MSCs detection verifies that circulating MSCs are obviously increased in various cancer patients [110, 111], indicating the mobilization of MSCs during tumorigenesis. In addition to recruiting distal MSCs, resident MSCs are another important source for TA-MSCs [11, 112]. Endometriosis-derived MSCs with low expression of CD10 are very similar to ovarian clear cell carcinoma-MSCs in phenotypes and tumor-promoting functions. Ovarian clear cell carcinoma efficiently educate endometriosis-derived MSCs [112]. Whether recruited MSCs or resident MSCs, MSCs derived from distinct tissues exhibit different tropisms towards the same type of tumor [113]. Additionally, cancer stem cells (CSCs) have a stronger capacity to recruit MSCs [114, 115] and MSCs display increased homing capacity to metastatic sites relative to primary tumors [116]. Furthermore, therapy strategies, including androgen deprivation for prostate cancer [117], irradiation for glioma [118] and breast cancer [119], as well as chemotherapy for breast cancer [120], remarkably increase the tropism of MSCs towards tumors and trigger the transition of resident MSCs into TA-MSCs. These data suggest that the extent of MSCs recruited and educated by tumor cells dynamically changes along with the malignant progression of tumors, thereby leading to alterations in the composition of TA-MSCs over time.

The diversity of cellular origin is also reflected in the extrinsic signaling-driven MSC tropism towards tumor sites. CXCL12/CXCR4 is the most classic signaling pathway widely involved in the recruitment of MSCs to tumors. CXCR4 expression levels influence the chemotactic ability of MSCs derived from various tissues, such as adipose, umbilical cord, amniotic membrane, and chorion, towards cervical cancer cells [113]. Irradiated cancer cells secrete elevated levels of CCL2, enhancing MSC migration to tumor sites via CCR2 [119, 120]. Additionally, various tumor cells have specific secreted profiles that recruit MSCs through distinct signaling pathways. Cytokine array analysis identifies CCL15 as being highly released by HCC cell lines. The CCL15-CCR1 pathway facilitates MSC migration and targeting to HCC both in vitro and in vivo [121]. Advanced renal cell carcinoma increases the secretion of AREG, FN1 and hyaluronic acid (HA), which enhance MSC migration and homing via CD44 [122]. MSCs are preferentially attracted to metastatic sites because HCC at these locations releases higher levels of epidermal growth factor (EGF), CXCL9, CCL25, and MMP9 [116]. Moreover, a previous study has demonstrated that intrinsic MMP1/its cognate receptor PAR1 axis is critical for MSCs migration and incorporation into glioma [123]. Notably, MMP1-mediated MSCs tumor tropism depends on interaction with extrinsic signaling CXCL12/CXCR4 [124]. This finding indicates that although pro-migration signals are crucial in MSC chemotaxis, the signals released by tumor cells may be the key determinants guiding their recruitment to tumors.

Naïve MSCs have long been considered direct cell sources of TA-MSCs due to their high similarity in basic biological characteristics. TA-MSCs may also derive from other cell types, including mesothelial cells and tumor cells. Verardo *et al. *found that HG-SOC-MSCs had a differential gene expression profile most similar to primary mesothelial cells and their derived cells. HG-SOC-MSCs exhibited a morphological change and expressed the epithelial cell marker keratin 7, similar to mesothelial cells, when cultured in a specified medium. Analysis of differential gene peaks usage data indicates that HG-SOC-MSCs likely originate from mesothelial cells [60]. MSC-like cells have been isolated and identified from colon cancer stem cells [125], implying that cancer cells might play a role in producing TA-MSCs. Genomic sequencing data also indicates that a minority of TA-MSCs might originate from glioma stem cells [38].

### Plasticity

Upon engraftment into tumors, naïve MSCs readily interact with the surrounding TME and undergo a phenotypic transition to heterogeneous TA-MSCs. This transition is highly stable and not driven by genetic mutations but rather tightly regulated by epigenomic mechanisms [59].

#### Transition into pro-inflammatory TA-MSCs

The concept of pro-inflammatory MSCs suggests that MSCs can be effectively recruited by a range of inflammatory mediators secreted by tumors, acquiring a pro-tumorigenic phenotype and function while preserving the essential characteristics of the original MSCs. IL-1β is commonly secreted by various tumor cells and triggers a pro-inflammatory reaction in naïve MSCs through the FAK and MAPK signaling pathways, albeit negatively modulated by TGFβ signaling [126]. Conversely, TGFβ is pivotal for osteosarcoma cells-conditioned medium (CM) inducing pro-tumor cytokines secretion by naïve MSCs [127]. This discrepancy is likely attributed to variations in tumor types, as specific TA-MSC generation is determined by the tumor cells with which they interact [40]. Functionally, ovarian tumor cell-CM induces the transition of MSCs to TA-MSCs by upregulating genes associated with chemotherapy resistance and enhancing the secretion of CXCL1, CXCL2, and IL-8 to suppress antitumor immunity [62]. IL-37 is critical for ovarian tumor cells to stimulate MSCs to express pro-tumor cytokines and pro-angiogenic factors [128]. Furthermore, multiple secreted chemokines and their corresponding receptors synergistically mediate this process. The interaction between CXCL6 secreted by breast cancer cells and CXCR6 on naïve MSCs leads to the induction of CXCL10 secretion by MSCs, which in turn acts on CCR3 on breast cancer cells, facilitating the recruitment of MSCs and promoting breast cancer metastasis. Concomitantly, MSCs-derived CCL5 binding to CCR5 on breast cancer cells result in the secretion of CSF1, which in turn binds to the CSF1 receptor on MSCs to recruit tumor-associated immune cells [129]. Non-coding RNAs are also involved, as evidenced by gastric cancer cell-CM promoting naïve MSCs transition into TA-MSCs by activating miR-155-5p-NF-κB signaling [130]. Tumor cell-derived extracellular vesicles (EVs) have been shown to induce inflammatory phenotypes in MSCs across various cancers, including neuroblastoma [131], lung tumor [132] and gastric cancer [21, 133]. Specifically, TA-MSCs with inflammatory features induced by osteosarcoma cells-EVs are essential for the development of drug resistance [134].

#### Trans-differentiation into cancer-associated fibroblast (CAF)-like cells

Different from the aforementioned inflammatory TA-MSCs, numerous studies suggest that MSCs are prone to transdifferentiate into CAF-like cells within TME. The initial evidence of direct MSC conversion into CAF-like cells was observed following prolonged exposure to supernatants from breast cancer, glioma, and pancreatic cancer cells. Microarray analysis and functional studies revealed upregulation of CAF-related genes in MSCs cultured in tumor cell-CM, accompanied by the acquisition of tumor-promoting functions [135]. Subsequent studies have consistently reported MSCs as significant contributors to CAF populations in various tumors, including colon cancer [136, 137], ovarian cancer [138, 139], prostate cancer [140, 141], and others. Soluble factors from tumor cells, including lysophosphatidic acid [142–144], CXCL16 [140], Wnt7a [138], osteopontin [145], HOXA9 [139], and EVs [146–150], are involved in promoting MSC trans-differentiation into CAF-like cells via various signaling pathways such as TGF-β/Smad, miRNAs, STAT3, MAPK, and integrins. Notably, both paracrine and autocrine TGF-β1 signaling play crucial roles in driving MSC adoption of CAF-like phenotypes [141, 145]. Analysis of the involved signaling pathways highlights the ubiquitous involvement of TGF-β/Smad signaling in this trans-differentiation process. Continuous exposure to pro-inflammatory cytokine, such as tumor necrosis factor α (TNFα) and IL-1β, effectively transforms MSCs into CAF-like cells [151]. Moreover, tumor-associated neutrophils induce MSC transformation into CAFs through secretion of pro-inflammatory cytokines, including IL-17, IL-23, and TNF-α [152], underscoring the role of chronic inflammatory microenvironments in this transition. Furthermore, increased acidity and ECM stiffness are identified as critical chemical and mechanical stimuli that expedite MSC trans-differentiation into CAF-like cells [153, 154]. Metabolic and epigenomic reprogramming also contribute to CAF-like differentiation of MSCs, as evidenced by shifts from oxidative phosphorylation (OXPHOS) to glycolysis [155] and metabolic alterations induced by factors such as pancreatic ductal adenocarcinoma-derived lactate [156]. Furthermore, a study highlighted that the transformation of MSCs into CAFs is contingent upon the expression levels of stem cell mark gene CD44 [157]. Notably, one study reported that CAFs derived from MSCs lacked PDGFRα expression, whereas resident CAFs did express it. In vivo bone marrow transplantation analyses reveal that MSC-derived CAFs replace resident CAFs, becoming the predominant fibroblast population within tumors [107]. This observation suggests that MSC-derived CAFs may exhibit distinct characteristics compared to the resident CAFs. While numerous studies have employed established CAF markers like α-SMA, vimentin, FAP, and SDF-1 to evaluate MSC differentiation into CAFs, it’s crucial to recognize that CAFs do not possess definitive markers, and these indicators may not fully capture CAF identity. Besides, α-SMA as a typical marker of myofibroblasts is commonly used to identify CAFs. MSCs exposed to prostate cancer-CM more resemble pro-inflammatory TA-MSCs rather than CAF-like cells due to reduced percentage of FAP and α-SMA double positive cells in these educated MSCs [158]. However, combined with the findings from a recent study regarding transition of MSCs into two classic subtypes of CAFs, including the inflammatory subtype and the myofibroblast subtype, we infer that pro-inflammatory TA-MSCs and CAF-like cells are probably the two main subtypes of TA-MSCs [159]. Further research is required to assess the interconvertibility of these subtypes and clarify their functional diversity within the TME.

#### Transition into other cell types

MSCs are capable to transit into the other stromal cells like endothelial cells [108, 160] and pericytes [161] within the TME. TNF-α has been observed to induce the differentiation of MSCs into endothelial cells via JNK-EGR1-VEGF2 signaling pathways in vitro. In an in vivo study, wild-type MSCs and EGR1-deficient MSCs were separately prepared from wild-type (Egr1^+/+^) and Egr1-null (Egr1^−/−^) mice, and then co-injected subcutaneously with 4T1 mouse breast cancer cells. Compared to the EGR1-deficient group and the 4T1 alone group, the wild-type group exhibited obvious positive staining for CD31, indicating endothelial cell differentiation [160]. Tissue-resident MSCs are typically located in the perivascular niche and are closely associated with pericytes [162]. Research supports the claim that local MSCs act as pericytes, facilitating the extravasation of melanoma cancer cells to distant metastatic locations such as the bone marrow and liver [163]. It has been demonstrated that platelet-derived growth factor-B (PDGF-B) secreted from tumor cells promotes the differentiation of MSCs into pericytes via interaction with neuropilin-1 [161]. Furthermore, in a chronic *H. pylori* infection mouse model, GFP-labeled MSCs were found to increase gastric cancer incidence. Co-expression of GFP and pan-cytokeratin cells in the mucosal layer indicate MSCs’ potential to differentiate into epithelial cells within the TME [10].

### Phenotype

Distinct gene expression profiles and secreted factors identified in TA-MSCs from various tumors, as well as from different patients with the same tumor type, directly demonstrate the heterogeneous phenotypes of TA-MSCs [40, 50]. The aforementioned heterogeneous cell origin and plasticity are also instrumental in understanding that TA-MSCs are composed of MSCs with varying phenotypes. Herein, we illustrate TA-MSCs with heterogeneous phenotypes within the same type of tumors. It is well-known that several classic surface antigens such as CD90, CD44, CD105 and CD73 are frequently used to characterize TA-MSCs. Flow cytometry analysis from numerous studies reveals that not all TA-MSCs express these markers, highlighting heterogeneity within TA-MSCs. CD90, in particular, has been extensively studied as a significant indicator of glioma-TA-MSC heterogeneity [164, 165]. Additionally, other genes contribute to TA-MSC heterogeneity, as evidenced by the presence of both GD2 positive and negative subpopulations within neuroblastoma TA-MSCs [100, 166]. Moreover, TA-MSCs from different patients may express GD2 but show heterogeneous expression of the macrophage checkpoint CD47 [70], suggesting diverse TA-MSC phenotypes among individuals. Furthermore, TA-MSC phenotypes can also vary across different tumor stages, as demonstrated by distinct proteomic signatures observed in low-grade glioma-derived TA-MSCs compared to those from high-grade gliomas [167]. Recently, scRNA-seq technology has become the preferred method for understanding cell heterogeneity at the single-cell level. scRNA-seq analysis directly confirmed the distinct phenotypes of CD90^+^ MSCs derived from primary breast cancer and normal tissues. The MSC population was divided into seven distinct clusters (C1-C7). The phenotypic identity of each cluster was determined based on gene expression associated with MSC-specific lineages and tissue origin, ultimately identifying them as osteogenic, adipogenic, PVL1, chondrogenic, myofibroblast-like, cycling, and patient-specific individual subpopulations [87]. Further validation of these distinct clusters is necessary to assess their association with pro-inflammatory TA-MSCs and CAF-like cells.

### Function

TA-MSCs exhibit heterogeneous phenotypes that often correspond to different functions. In breast cancer-TA-MSCs, a patient-specific cluster was identified to express a unique set of genes closely associated with ECM remodeling [87], suggesting their distinct function from the remain clusters. Differential gene expression and secreted factors observed between CD90^−^ and CD90^+^ glioma-TA-MSCs indicate their divergent roles in glioma progression [164]. Experiments conducted by Zhang *et al.* indeed confirm that the CD90^−^ subpopulation mainly boost glioma cell proliferation, migration, and adhesion, whereas the CD90^+^ subpopulation primarily facilitates neoangiogenesis via pericyte differentiation and interaction with endothelial cells [168]. Furthermore, the functional heterogeneity of TA-MSCs may be linked to their diverse plasticity. As previously discussed, if pro-inflammatory TA-MSCs and CAF-like cells represent the two primary types of TA-MSCs, they are likely to synergistically promote tumor progression by modulating tumor immunity and remodeling the ECM, respectively.

Functional heterogeneity are also observed in MSCs derived from different sites. Differential protein expression profiles identified between TA-MSCs originating from low-grade and high-grade gliomas indicate potential functional distinctions between these two types of TA-MSCs [167]. TA-MSCs derived from primary tumor sites primarily promote cancer cell proliferation and dissemination, while those from metastatic sites predominantly stimulate cancer cell re-seeding [48]. Inconsistent functions of TA-MSCs have also been observed among different patients [50]. Interestingly, Berger* et al.* simultaneously isolated tumor cells and MSCs from clinical gastric carcinoma (GSC) and lung carcinoma (LC) tissues, demonstrating that only GSC-derived MSCs, were crucial for promoting GSC proliferation. Mechanistically, GSC-MSCs exhibited elevated hepatocyte growth factor (HGF) levels and higher expression of genes that are favorable for tumor cell proliferation and migration compared to LC-MSCs [40].

It should also be noted that the observed inconsistent functions of TA-MSCs sometimes are more likely caused by experimental conditions. Among these conditions, the direct or indirect co-culture system has been recognized as a significant factor leading to different or even opposite effects of TA-MSCs on tumor cells [45, 47, 169, 170]. Furthermore, the choice between 2D or 3D co-culture models within the direct coculture system can also result in divergent regulatory outcomes [171]. Additionally, a recent study revealed that neuroblastoma-TA-MSCs at low passages exhibit increased proliferative activity and were nearly negative for senescence-associated β-galactosidase staining. In contrast, TA-MSCs at high passages displayed contrasting results. Consequently, TA-MSCs at low and high passages were designated as young and senescent TA-MSCs, respectively. Remarkably, the young TA-MSCs, rather than the senescent ones, significantly suppressed the proliferation and immunity of NK cells [70]. From this point of view, the number of passages should also be taken into account when studying TA-MSCs.

## Tumorigenic roles and mechanisms of TA-MSCS in tumor evolution

**Fig. 3:**
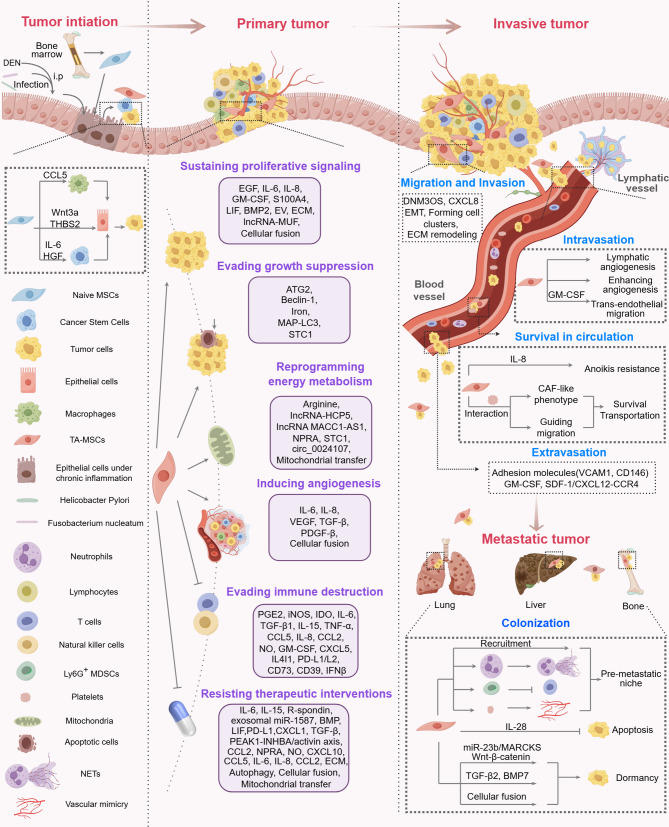
Tumorigenic roles and mechanisms of TA-MSCs in tumor evolution. Tumor evolution is roughly divided into four stages including tumor initiation, primary tumor, invasive tumor and metastatic tumor. MSCs activated by chronic inflammation are crucial in the initiation of tumorigenesis, as evidenced by various models of chronic inflammation-induced carcinogenesis. These cells also contribute to genetic instability and may facilitate the emergence of CSCs through the release of soluble factors. During the primary tumor stage, TA-MSCs support tumor growth and survival by modulating several hallmark capabilities. Additionally, TA-MSCs enhance the dissemination of tumor cells by promoting their migration, invasion, intravasation, survival in circulation, and extravasation. Upon reaching distant tissues, MSCs are actively recruited to participate in the formation of the pre-metastatic niche, where they confer resistance to apoptosis and induce dormancy in tumor cells, thus facilitating their colonization and the establishment of distal metastases

Tumorigenesis and progression involve complex events where tumor cells gain key capabilities for growth and spread [172]. Kye processes include maintaining proliferative signaling, resisting cell death, avoiding growth suppression, achieving replicative immortality, escaping immune destruction, altering energy metabolism, enhancing angiogenesis, and facilitating invasion and metastasis [172]. While the tumor-promoting effects of MSCs have been extensively reviewed in existing literature [15, 173, 174], a systematic analysis that integrates cancer hallmark capabilities to elucidate the comprehensive roles of TA-MSCs throughout the entire tumor progression continuum from initiation to metastasis remains absent. This section outlines the roles and mechanisms of TA-MSCs in tumor progression from initiation to metastasis through modulation of these capabilities (Fig. 3).

### Tumor initiation

Chronic inflammation is recognized as a significant factor contributing to the development of various tumors. Intriguingly, it has been uncovered that MSCs are participated in tumorigenesis within the context of chronic inflammatory microenvironments. In a liver cancer model induced by DEN exposure, AIF1^+^CSF1R^+^ MSCs exhibiting elevated expression of sirtuin 1 (SIRT1) was identified within liver tissues undergoing chronic inflammation prior to the onset of cancer. MSCs overexpressing SIRT1 significantly expedited cancer initiation and progression by secreting CCL5, which enhanced macrophage recruitment and amplified the inflammatory response [11]. Similarly, a three-month infection with *H. pylori* followed by the transplantation of BM-MSCs into the stomach of mice resulted in a marked increase in the incidence of intraepithelial neoplasia and gastric cancer. Mechanistically, *H. pylori*-induced chronic inflammation promoted BM-MSC migration, their transition into epithelial cells and CAFs, as well as secretion of Thrombospondin-2 (THBS2) [10]. In Apc^Min+^ mice with chronic Fusobacterium nucleatum infection, BM-MSCs migrate to the mucosal layer and promote colorectal carcinogenesis by secreting Wnt3a, which activates the Wnt/β-catenin/TGIF signaling pathway [175]. Furthermore, Rubinstein-Achiasaf *et al.* noted that prolonged exposure to the inflammatory cytokines TNF-α and IL-1β can transform MSCs into oncogenic inflammatory CAFs [151]. These transformed MSCs secrete inflammatory factors that promote tumorigenesis, implying that MSCs under chronic inflammatory conditions may facilitate epithelial cells in evading immune surveillance, proliferating uncontrollably, resisting apoptosis, and attaining cellular immortality.

A prevailing view in tumorigenesis posits that cancer arises from the accumulation of genetic mutations, with CSCs being pivotal in tumor initiation. CSCs, which share many characteristics with normal stem cells, are thought to undergo malignant transformation from normal stem cells as a result of oncogenic mutations [176, 177]. MSCs selectively enrich CSCs in colon adenoma and melanoma cells by secreting IL-6 and HGF. Subsequent cytogenetic analyses have revealed alterations in chromosome 17 (17q25), where stem cell-related genes and microRNAs are located, in tumor cells cultured in MSCs-CM [178]. This study indicates that the secretome derived from MSCs may facilitate the generation of CSCs by influencing genetic stability. Although few studies have reported that MSCs induce genetic instability in normal epithelial cells, it is hypothesized that as chronic inflammation progresses, stem cells or epithelial cells may become increasingly susceptible to genetic alterations induced by MSCs from chronically inflamed tissue.

### Primary tumor growth and survival

At present, most studies have concentrated on elucidating the regulatory functions and mechanisms of TA-MSCs in relation to primary tumors. TA-MSCs significantly influence the following hallmark capabilities to promote the progression of primary tumors.

#### Sustaining proliferative signaling

Research consistently shows that TA-MSCs promote tumor cell proliferation through various mechanisms. One primary mechanism involves the secretion of soluble factors such as EGF [18], IL-6 [38, 58, 179], IL-8 [42, 44], granulocyte-macrophage colony-stimulating factor (GM-CSF) [78], S100A4 [66], leukemia inhibitory factor (LIF) [179], and BMP2 [61]. These factors have been identified as mediators that activate pathways linked to cell proliferation and stemness in tumor cells. Additionally, TA-MSC-derived EVs deliver essential cargos such as miR-221 and LINC01559 to favor tumor growth [93, 180]. Furthermore, ECM components enhance MSC communication with tumor cells to augment tumor proliferation [181]. A secondary mechanism involves TA-MSCs directly influencing critical regulators in tumor cells to increase their proliferative activity. In HCC, TA-MSCs enhance tumor development and growth by increasing the expression of long non-coding RNA (lncRNA)-MUF in tumor cells. LncRNA-MUF activates Wnt/β-catenin signaling and EMT by binding to Annexin A2 (ANXA2) and sponges miR-34a to promote upregulation of Snail1 and EMT [65]. While certain studies indicate that MSCs rarely or never fuse with tumor cells [182, 183], it has been evidenced that TA-MSCs can enhance tumor cell proliferation via cellular fusion [184, 185].

#### Evading growth suppression

As the size of a tumor increases, the tumor’s core frequently encounters nutritional deficiencies. In response to this, TA-MSCs have been observed to engage in autophagy to ensure their own survival, while simultaneously releasing factors that protect tumor cells from apoptosis [106, 186]. Notably, in the context of ovarian clear cell carcinoma, cellular proliferation is reliant on iron availability. CD10-negative endometriosis-derived MSCs have been proven to provide iron to tumor cells in co-culture systems, thereby mitigating the growth suppression effects induced by iron chelation [112]. TA-MSCs derived from gastric cancer can effectively negate the inhibitory effects of peripheral blood mononuclear cells (PBMCs) on gastric cancer growth in vivo. This is achieved through modulation of the Treg/Th17 cell ratio [187]. MSCs co-cultured with lung cancer cells can protect these cancer cells from ROS-induced apoptosis by regulating mitochondrial respiration [188].

#### Reprogramming energy metabolism

Tumor cells adapt to harsh conditions through metabolic reprogramming, with TA-MSCs playing a key role in enhancing their metabolic flexibility. Nitric oxide (NO) is endogenously synthesized by nitric oxide synthase (NOS) through the conversion of l-arginine to citrulline. TA-MSCs enhance NO synthesis in ovarian and endometrial cancer cells by secreting arginine, following arginine depletion with l-arginase and NOS inhibition with l-NAME. This process promotes glycolysis and reduces oxidative stress, aiding tumor growth [189]. This suggests metabolic coupling between TA-MSCs and tumor cells in maintaining NO balance. MSCs co-culture promotes therapeutic resistance in estrogen receptor-positive (ER^+^) breast cancer cells by enhancing oxidative phosphorylation and increasing intracellular ATP levels [190]. Similarly, co-culture with lung cancer cells prompts MSCs to secrete stanniocalcin-1 (STC1), enhancing the survival of lung cancer cells by shifting their metabolism from oxidative phosphorylation to a more glycolytic state [188]. Furthermore, in a gastric cancer cell co-culture model, MSCs upregulate lncRNA HCP5 [191], natriuretic peptide receptor A (NPRA) [192], and lncRNA MACC1-AS [193] in cancer cells, promoting fatty acid oxidation (FAO)-mediated stemness and chemoresistance. Likewise, gastric cancer-associated TA-MSCs stimulate lymphatic metastasis by activating FAO in cancer cells via circ_0024107 [102]. Mitochondria are essential for oxidative metabolism in eukaryotes. A more recent study indicates that transferring mitochondria to tumor cells with low mitochondrial content is a new way that ovarian cancer TA-MSCs promote tumor progression [194].

#### Inducing angiogenesis

Angiogenesis is crucial for enabling tumor cells to obtain essential nutrients and oxygen while facilitating the removal of metabolic waste products and carbon dioxide. Increasing evidence suggests that TA-MSCs promote angiogenesis through four primary mechanisms: First, TA-MSCs from various tumors consistently show increased levels of pro-angiogenic factors like VEGF and IL-6 [19, 42, 66, 68]. The *H. pylori*-induced gastric cancer mouse model reveals that MSCs acquire the capability to enhance tumor neovascularization upon recruitment to the tumor microenvironment [195]. Second, it has been demonstrated that the interaction between MSCs and tumor cells augments the secretion and expression of angiogenesis-promoting factors by both cell types [42, 50, 66, 91, 106, 143, 196]. Interestingly, some investigations have demonstrated that MSCs can fuse with glioma cells, thereby increasing the stemness and angiogenic potential of the tumor cells [197]. Third, TA-MSCs enhance tumor vascularization by stimulating adjacent macrophages to produce pro-angiogenic factors like IL-6, IL-8, and VEGF [198]. Finally, TA-MSCs directly contribute to vessel formation through cellular transdifferentiation. These cells can differentiate into endothelial cells or pericytes-a process enhanced by p38α deficiency through TGF-β/JNK pathway activation in colon cancer [199]. Tumor cell-secreted PDGF-B converts MSCs into pericytes and recruits them via interaction with neuropilin-1, thus promoting angiogenesis [161].

#### Evading immune destruction

It is well-documented that TA-MSCs secrete a variety of immunomodulatory mediators, including prostaglandin E2 (PGE2), inducible nitric oxide synthase (iNOS)/indoleamine 2,3-dioxygenase (IDO), TGF-β1, and IL-6, to exert their immunomodulatory effects. TA-MSCs orchestrate a complex immunosuppressive network through cell-type-specific mechanisms that target both myeloid and lymphoid lineages [14, 15, 173, 200]. Within the myeloid compartment, macrophages are modulated and recruited by TA-MSCs via soluble inflammatory cytokines and chemokines such as TGF-β1, IL-6 [201], TNF-α, CCL5 [11], and CCL2 [202]. Macrophages are further polarized into the M2 phenotype and organized into immunosuppressive niches that exclude CD8^ +^ T cells, as demonstrated by scRNA-seq analysis in ovarian cancer [203]. Neutrophils are functionally reprogrammed by TA-MSCs through IL-6-STAT3-ERK1/2-mediated apoptosis inhibition and pro-tumor activation in gastric cancer [204], while myeloid-derived suppressor cells (MDSCs) are expanded by TA-MSCs via CXCL5/NO/GM-CSF secretion in lung cancer models [205]. TA-MSCs disrupt dendritic cells (DCs) metabolism through cystathionase suppression in melanoma, thereby impairing T cell proliferation and anti-tumor efficacy [206]. Within the lymphoid compartment, NK cells undergo functional impairment as a result of the transition of CD56^dim^ cells and the suppression of IFN-γ in the CD56^bright^ subset, which is induced by TA-MSCs-originated PGE2 and IL-6 in lung squamous carcinoma [47]. T cells are systematically inhibited by TA-MSCs via multiple pathways: IDO [33, 207] and PD-L1/L2 pathway [39, 82] facilitate direct suppression; the CD39/CD73-adenosine axis [91, 94], sialylation-mediated PD-1 and Siglec-7/Siglec-9 expression [208], and PD-L1-dependent granzyme B inhibition [209] contributes to CD8 + T cell exhaustion; and IL-15-STAT5 signaling promotes the expansion of Tregs while inhibiting Th17 cells [187, 210, 211]. B cells encounter a blockade in proliferation through direct contact with TA-MSCs [212] and IDO-dependent inhibition of infiltration [207], with disruption of tertiary lymphoid structures by suppressing B cell proliferation observed in HG-SOCs [213, 214]. Additionally, TA-MSCs enhance immunosuppression by altering tumor cell immunogenicity through the downregulation of HLA-I [95, 215], upregulation of PD-L1 via IL-8 secretion [96, 216], and increased expression of CD73 [91]. Taken together, this multidimensional immunosuppressive network, spanning myeloid cell reprogramming and lymphoid cell dysfunction, highlights TA-MSCs’ role as master regulators of tumor immune evasion.

#### Resisting therapeutic interventions

Therapy resistance continues to pose a significant clinical challenge in the treatment of tumors. Emerging evidence indicates that TA-MSCs contribute to therapy resistance through a variety of mechanisms: (I) Enhancing the stemness of tumor cells: Cross-talk between MSCs and tumor cells facilitates CSC niche formation [217, 218]. TA-MSCs from tumors like osteosarcoma [219], breast cancer [220], ameloblastoma [68], gastric cancer [211, 221], glioma [38, 98], and ovarian carcinoma [61, 179], promote tumor cell stemness by releasing factors such as IL-6, IL-15, R-spondin, exosomal miR-1587, BMP, and LIF. Although these investigations did not directly assess whether tumor cells conditioned by TA-MSCs acquired therapy resistance, the enhancement of stemness implies that tumor cells may be better equipped to evade therapeutic elimination. Concurrently, other studies have explicitly demonstrated that TA-MSCs enhance tumor cell stemness, thereby conferring resistance to therapy. For instance, gastric cancer-TA-MSCs stimulate CSC properties by upregulating PD-L1 in cancer cells, reducing sensitivity to fluorouracil (5-FU) and paclitaxel [43, 44, 222]. Breast cancer-TA-MSCs secrete CXCL1 to induce doxorubicin resistance via upregulation of ABCG2 in triple-negative breast cancer [223]. Additionally, another study has uncovered that MSCs promote breast cancer cells’ acquisition of a CSC-like phenotype to resist antiestrogenic drug therapy [224]. (II) Activating cell survival pathway to suppress apoptosis: Ovarian carcinoma-TA-MSCs protect cancer cells from carboplatin-induced apoptosis through activating PI3K-AKT pathway and phosphorylation of X-linked inhibitor of apoptosis protein (XIAP) [225]. Likewise, gastrointestinal stromal tumor-TA-MSCs promote imatinib mesylate resistance through secretion of TGF-β to activate PI3K-AKT pathway [84]. PYK2 activation is pivotal for ovarian cancer cell growth and survival [226]. Ovarian cancer-TA-MSCs-derived CCL2 and CCL5 increase IL-6 expression in cancer cells, promoting chemotherapy resistance via PYK2 phosphorylation [25]. A novel PEAK1-INHBA/activin axis has been recently revealed to mediate TA-MSCs to potentiate lapatinib resistance in HER2-positive breast cancer through anti-apoptotic and stress signaling pathways [227]. (III) Modulating metabolism remodeling to drive therapy resistance: MSCs have been found to upregulate gastric cancer cell expression of NPRA, thus increasing FAO activity to resist cisplatin therapy [192]. Similarly, AD-MSCs contribute to paclitaxel resistance in endometrial cancer cells by elevating NO synthesis, which boosts glycolysis and reduces oxidative stress [189]. A recent study highlights MSCs’ influence on tumor cell metabolic plasticity, proposing that targeting metabolic pathways in ER^+^ breast cancer cells could effectively address their resistance to conventional treatments [190]. Interestingly, in a coculture system, MSCs are capable to increase gastric cancer cells’ resistance to oxaliplatin through transferring mitochondria [228]. (IV) MSCs educated by therapeutic drugs: TA-MSCs exhibit higher resistance to chemotherapy than tumor cells and could be educated by chemotherapeutic drugs to potentiate therapy resistance [229]. For example, gemcitabine chemotherapy educates MSCs to release CXCL10 to enrich CSC [230]. Pre-exposure to cisplatin induces apoptosis resistance and senescence-associated phenotype in MSCs, while also increasing the secretion of IL-6, IL-8 and other cytokines, thereby enhancing the chemoresistance and stemness of tumor cells [229]. The anti-tumor drug 3,3’-diindolylmethane (DIM) activates NF-κB signaling in gastric cancer-TA-MSCs, enhancing their secretion of IL-6, IL-8 and CCL2, which in turn promotes cancer cell proliferation [231]. Beyond the aforementioned mechanisms, other mechanisms including ECM components [232], autophagy activation [51], and hybrid cell formation [233] have been explored as potential mediators of TA-MSCs-driven chemoresistance.

### Tumor metastasis

Tumor metastasis is a complex, multi-step process where primary tumor cells migrate and invade nearby tissues, intravasate into the circulatory system, survive in circulation, extravasate into distant tissues, and eventual colonize in their parenchyma [234–236]. Accumulative evidence indicates that TA-MSCs significantly influence multiple stages of the invasion-metastasis cascade [78, 237].

#### Enhancing cellular migration and invasion

The ability to migrate and invade is essential for tumor cells to depart from primary sites and initiate metastasis. A myriad of studies underscore TA-MSCs’ role in augmenting tumor cell migratory and invasive capacities through their secretome. HCC-TA-MSCs-CM upregulates lncRNA DNM3OS in tumor cells, accelerating their migration and invasion [67]. IL-8 has been identified to separately mediate oral squamous cell carcinoma-TA-MSCs and gastric cancer-TA-MSCs to enhance tumor cell motility and invasion [36, 97]. However, many studies didn’t clarify the underlying regulatory mechanisms. The EMT program is recognized for imparting tumor cells with crucial properties for invasion and metastasis, such as enhanced motility, invasiveness, and ECM degradation. TA-MSCs from various tumor tissues, including lung cancer [45, 50], HCC [65], prostate cancer [140], ameloblastoma [68] and gastric cancer [41, 211], have been shown to directly or indirectly trigger tumor cell EMT. Additionally, fusion with or engulfment by MSCs represents significant mechanisms fostering tumor cell EMT and enhancing metastatic potential [238–240]. The increased expression of lysyl oxidase (LOX) in breast cancer cells facilitates MSC-induced EMT without impacting stemness [241]. Intriguingly, Zarubova *et al.* recently have observed that MSCs cluster with tumor cells both in vitro and in vivo, facilitating tumor cell dissemination. They metaphorically described MSCs as cellular taxis for this phenomenon [242]. Microfluidic devices have demonstrated that MSCs can direct cancer cell movement in a cluster-sprout-infiltration pattern, underscoring their role as carriers in tumor cell migration [243]. Moreover, TA-MSCs can produce ECM proteins and related enzymes that change tissue architecture and composition, thus promoting the invasive phenotype of tumor cells [50, 144, 244, 245].

#### Intravasation and survival in the circulation

Intravasation, where metastatic cells detach from the primary tumor to enter the vascular system, is heavily influenced by vascular density, as tumor cells necessitate vascular access to infiltrate the bloodstream. As discussed in the preceding section on “Inducing Angiogenesis”, TA-MSCs are well-documented promoters of angiogenesis. The newly formed blood vessels typically originate from pre-existing capillaries and exhibit abnormal structures and increased permeability. Consequently, the presence of TA-MSCs may predispose highly vascularized tumors to enhanced intravasation. Direct evidence supporting this phenomenon is provided by studies involving pancreatic cancer-associated TA-MSCs [78]. Co-inoculation of GFP-labeled tumor cells with RFP-labeled TA-MSCs into the pancreatic tail of NOD/SCID mice significantly increases the presence of GFP-positive tumor cells in the bloodstream compared to injecting tumor cells alone. TA-MSCs notably augment the trans-endothelial migration of tumor cells in vitro, primarily due to their secretion of GM-CSF. Lymphatic vessels offer an alternative route for tumor cell intravasation, indirectly facilitating access to the vasculature. It is crucial to recognize that the structural characteristics of blood vessels and lymphatic vessels differ significantly, with lymphatic vessels lacking the tight endothelial junction characteristic of blood vessels. Tumor cell entry into lymphatic vessels requires invasion through connective tissue, relying on lymphatic vasculature formation and the cells’ migratory and invasive abilities. Research on gastric cancer-TA-MSCs and MSCs educated by gastric cancer reveals their substantial role in enhancing lymphatic angiogenesis, tumor cell migration, and invasion, which int turn supports lymphatic metastasis [21, 102, 133].

Following intravasation, tumor cells encounter numerous challenges including exposure to blood flow shear stress, immune surveillance, and anoikis. To establish metastases, tumor cells must interact with various cell types to evade immune clearance and apoptosis. Anoikis is a form of cell death that triggers apoptosis to inhibit metastasis when tumor cells detach from their ECM or neighboring cells. Studies have reported that MSCs produce IL-8, promoting lung metastasis of osteosarcoma cells by conferring resistance to anoikis [246]. Besides, platelets activated by tumor cells not only induce MSCs adoption of CAF-like phenotype to potentiate gastric cancer lung metastasis [247] but also guide MSCs migration towards metastatic sites [81], indicating platelet involvement in creating niches for tumor cell survival and transportation. Studies involving the co-injection of tumor cells with MSCs into veins or spleens in animal models consistently demonstrated increased metastatic potential, implicating pivotal roles of MSCs in tumor dissemination [45, 106].

#### Extravasation

Extravasation necessitates the initial adhesion and subsequent arrest of tumor cells at the endothelial lumen. TA-MSCs derived from lung cancer and glioma exhibit an increased ability to promote tumor cell adhesion [144, 168], thereby potentially facilitating their attachment to endothelial cells. Likewise, MSCs educated by tumor cells display increased expression of adhesion molecules [248, 249]. These adhesion molecules serve not only to mediate MSC migration towards and adhesion to tumor cells [250, 251] but also to stimulate their adhesion to endothelial cells [244, 252]. Given the tendency of MSCs to form cell clusters with tumor cells, their adhesion to endothelial cells naturally promotes the attachment of tumor cells to the endothelium. Following adhesion, circulating tumor cells must exit the blood vessels, a process known as extravasation. Waghray *et al.* observed that TA-MSCs accompany tumor cells throughout their circulatory journey, ultimately traversing the blood vessel walls to reach the liver. Within the liver parenchyma, MSCs predominantly localize near tumor cells, with both cell types situated adjacent to blood vessels. The targeted knockdown of GM-CSF secretion from TA-MSCs completely abrogate this phenomenon, underscoring the critical role of TA-MSCs in this process [78]. In a mouse model of bone and liver metastasis initiated by melanoma cell infusion, MSCs have been shown to function similarly to pericytes at metastatic sites, and facilitate melanoma cell extravasation through the adhesion molecule CD146 and the SDF-1/CXCL12-CXCR4 signaling pathway [163].

#### Colonization at the distant sites

Colonization describes the capacity of tumor cells to persist and proliferate in secondary tissues or organs. Tumor metastasis is an active process. Prior to metastatic dissemination, primary tumors secret factors and release EVs to establish a pre-metastatic niche conducive to tumor cell colonization [253]. The successful isolation of MSC-like cells from metastatic lymph nodes and liver, along with their heightened abilities to promote tumor growth and metastasis, underscores the potential involvement of MSCs in pre-metastatic niche formation [20, 21].

As widely acknowledged, many tumors tend to metastasize to bone. The role of BM-MSCs in forming the pre-metastatic niche has been widely investigated. BM-MSCs have been shown to induce mesenchymal-to-epithelial transition (MET) of metastatic lung cancer cells, thereby fostering cancer cell proliferation and macro-colony formation in bones [48, 237]. Similarly, BM-MSCs facilitate breast cancer cells in forming bone metastases by modulating key hallmark capacities, including promoting angiogenesis, forming therapy resistance, and avoiding immune surveillance [254]. Furthermore, interleukin-28 secretion from BM-MSCs has been implicated in imparting resistance to apoptosis in prostate cancer cells, consequently facilitating bone metastasis [255]. Recently, it has been revealed that BM-MSCs co-cultured with breast cancer cells transfer GIV protein to the cancer cells via connexin 43-mediated tunneling nanotubes, thereby promoting cancer cell dissemination to the bone marrow [256].

Another prevalent site of metastasis is the lungs. Mouse lung tissue-resident MSCs, like BM-MSCs, have been shown to aid in creating a pre-metastatic niche that enhances cancer cell survival and proliferation. Mechanistically, they attract neutrophils, trigger neutrophil extracellular traps (NETs) formation, and promote neutral lipid accumulation in neutrophils, which are subsequently transferred to cancer cells [27, 257]. MSCs educated by tumor cells expand Ly6G^+^ MDSCs to dampen the anti-tumor immune response of T cells, thereby fostering an immunosuppressive pre-metastatic niche that shields disseminated lung cancer cells at metastatic sites [205]. In addition, tumor-activated platelets enhance MSC migration to the lungs, enabling them to form vascular mimicry (VM) and establish a pre-metastatic niche, which may facilitate tumor cell extravasation and survival [81].

Upon colonization of metastatic sites, tumor cells swiftly enter a state of dormancy, the duration of which remains uncertain. Emerging studies highlight the crucial role of BM-MSCs in driving dormancy in metastatic breast cancer cells through diverse mechanisms. BM-MSCs-derived EVs promote a dormant phenotype in metastatic breast cancer cells via the miR-23b/myristoylated alanine-rich C-kinase substrate (MARCKS) axis [258] and Wnt-β-catenin signaling-mediated dedifferentiation [259]. A specific subset of NG2^+^/Nestin^+^ BM-MSCs, known to promote hematopoietic stem cell quiescence, was discovered to induce dormancy in breast cancer cells by the release off TGFβ2 and BMP7. Disruption of dormancy and recurrence of bone metastasis were observed in the absence of MSCs or through conditional knockout of TGFβ2 [260]. Furthermore, although current evidence suggests low and unstable fusion events between tumor cells and MSCs, breast cancer cells have been shown to exploit this mechanism to acquire a dormant phenotype [261].

## Clinical implications of TA-MSCS

**Fig. 4:**
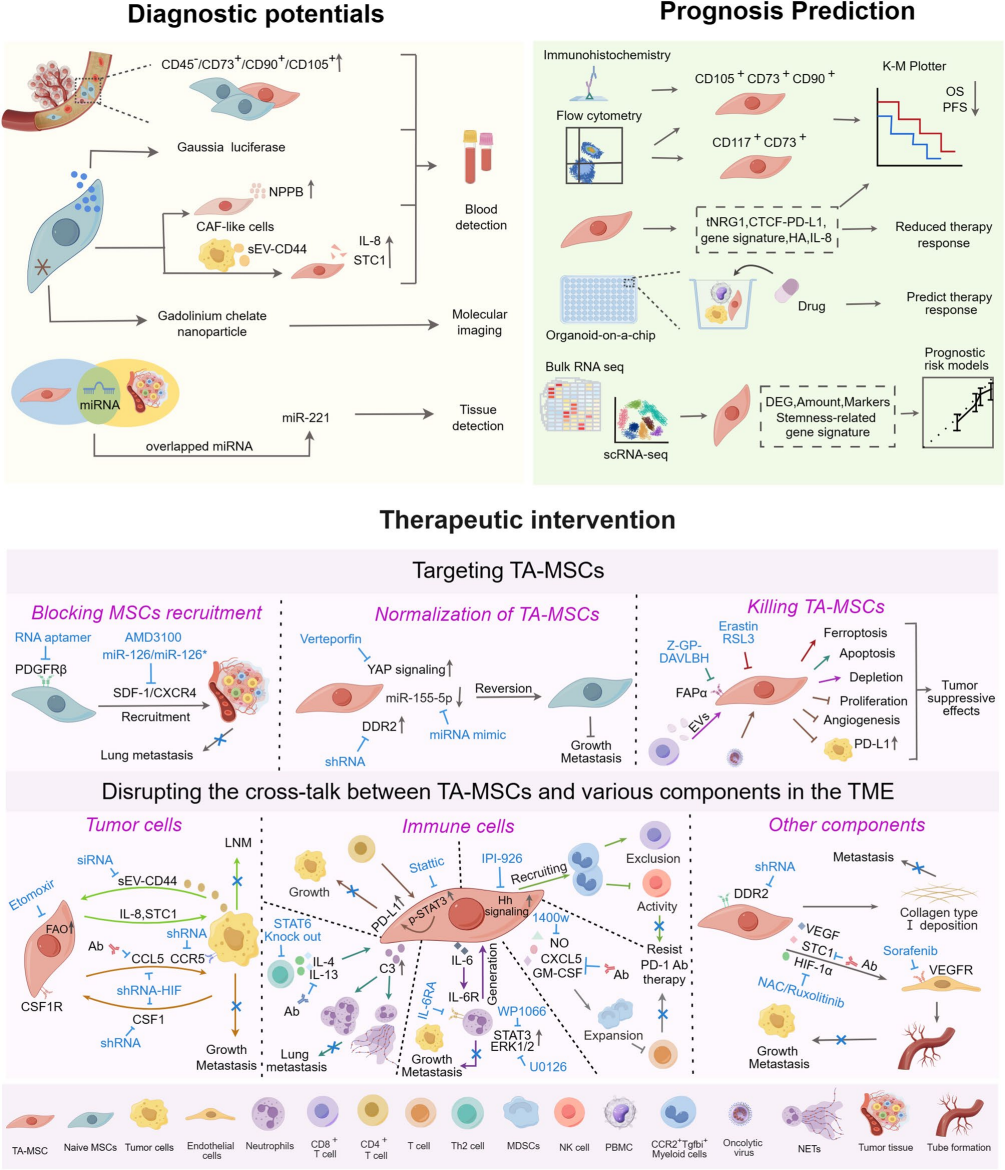
Clinical implications of TA-MSCs in tumor diagnosis, prognosis prediction and therapeutic interventions. Diagnostic Potential: TA-MSCs and their associated molecules hold promise as diagnostic tools for cancer detection through blood tests, molecular imaging, and tissue analysis. Prognostic Prediction: The quantification of TA-MSCs and their related molecules may serve as indicators of poorer prognosis and therapy response. The development of an HCC organoid-on-chip model, achieved by co-culturing MSCs with PBMCs, presents potential for improved prediction of immunotherapy responses. Prognostic risk models, constructed using differentially expressed genes (DEGs), quantities, and markers of TA-MSCs, are instrumental in forecasting prognosis and therapeutic outcomes. Therapeutic Intervention: Therapeutic intervention strategies encompass the direct targeting of TA-MSCs and the disruption of their intricate interactions with various components of the TME. The blue font indicates the method of intervention, while the symbol ‘X’ denotes blockade

The diverse roles of TA-MSCs in tumorigenesis are gaining recognition. Significant research has focused on understanding the molecular mechanisms behind their tumor-promoting effects. Studies also suggest TA-MSCs have potential in tumor diagnosis, prognosis, and therapy. This section explores their clinical implications in these three areas (Fig. 4).

### Diagnostic potentials

The well-documented tropism of naïve MSCs towards tumor sites is essential for the generation of TA-MSCs. Some studies have begun to harness this inherent tumor tropism property to investigate potential avenues for tumor diagnosis. One prospective biomarker study found significantly higher circulating levels of MSCs in the peripheral blood of cancer patients compared to healthy individuals [111]. More significantly, Liu *et al.* engineered “reporter MSCs” to produce humanized Gaussia luciferase (hGluc) and transplanted them into a mouse lung metastasis model. These MSCs exhibited prolonged residence in tumor-bearing lungs than in tumor-free lungs. Following MSC transplantation for 48 h, an increase in circulating hGluc activity was detected in the tumor-bearing group, persisting for 10 days, which indicates the potential utility of infused hGluc-secreting MSCs in developing a straightforward blood test for cancer detection [262]. Furthermore, MSCs labeled with various probes have been administered into murine tumor models, offering a potential method for cancer detection through tracking. This approach also represents a promising avenue for molecular imaging research [263–265].

Some studies also have explored TA-MSCs-associated molecules for diagnostic purposes. Epithelial ovarian cancers-CM efficiently induces CAF-like differentiation of MSCs. Microarray analysis was conducted to assess molecular alterations during the transition process, revealing prominent upregulation of natriuretic peptide B (*NPPB*) in the educated MSCs. Elevated NT-proBNP protein levels in peripheral blood of ovarian cancer patients without cardiac failure indicates that tumor stromal-derived NPPB could serve as a candidate biomarker for cancer diagnosis [266]. ROC curve analysis also demonstrated superior diagnostic performance of gene levels of critical modulators involved in the education of BM-MSCs by gastric cancer cell-derived exosomes [133]. Our previous analysis revealed overlapping upregulated miRNAs in both TA-MSCs and cancer tissues, highlighting miR-221 as a promising diagnostic marker for gastric cancer [93]. The findings indicate that integrating TA-MSCs with tumor tissue analysis could provide an effective strategy for identifying potential diagnostic markers.

### Prognosis prediction

It is well established that TA-MSCs exacerbate malignant progression, correlating with poorer prognosis and diminished therapeutic efficacy. Immunohistochemistry assays commonly utilize three classical surface antigens, CD105, CD73, and CD90, to identify gastric cancer stromal TA-MSCs. Patients with positive expression of these markers exhibit shorter overall survival (OS) compared to those with negative expression. Multivariate analyses revealed that CD105^+^ TA-MSCs serve as an independent prognostic factor in gastric cancer [267]. Likewise, flow cytometry quantification of glioma-TA-MSCs based on these markers reveals a negative association with OS across three cohorts of glioma patients [83]. CD117^+^ fibroblast-like stromal cells in ovarian cancer tissue show CD73 positivity, indicating they are TA-MSCs. These cells are linked to tumor progression and are inversely correlated with OS and progression-free survival (PFS) [268]. However, the relationship between TA-MSC abundance and therapeutic responses remains underexplored.

Increasing studies have concentrated on examining molecules linked to TA-MSCs and their impact on prognosis and therapeutic response. Transmembrane neuregulin 1 (tNRG1), expressed by colon cancer-associated TA-MSCs, has been positively correlated with advanced cancer stages and a reduced five-year PFS rate [269]. In gastric cancer, TA-MSCs increase cancer cell resistance to 5-FU therapy by upregulating CTCF-PD-L1, which is associated with diminished chemotherapy responses [43]. In breast cancer, CD10^+^ stromal cells, which likely originate from BM-MSCs, exhibit a gene signature associated with poor prognosis and non-response to chemotherapy in HER2-positive patients [270]. Additionally, MSCs have been found to secrete HA, contributing to breast cancer chemotherapy resistance, with serum HA levels positively correlating with chemoresistance [232]. Despite the promising potential of immunotherapy for various tumors, researchers are endeavoring to elucidate the mechanisms underlying its limited efficacy. Recent studies reveal the pivotal roles of TA-MSCs in contributing to immunotherapy resistance [192, 203, 216]. Gastric cancer-TA-MSCs release IL-8, enhancing PD-L1 expression and lactate production in cancer cells, thus impairing antitumor immunity and diminishing the efficacy of anti-PD-1 therapy [216]. Elevated serum IL-8 levels correlate with reduced efficacy of anti-PD-1 therapy [216, 271]. Beyond molecular investigations, a recent study co-cultured MSCs with PBMCs to develop HCC organoids-on-chip. This approach provides a more accurate prediction of immunotherapy response models compared to traditional organoid systems [272].

In addition to experimental evidences, a growing body of bioinformatics analysis further supports the potential implication of TA-MSCs in this aspect. A prognostic risk model utilizing differentially expressed genes (DEGs) in lung cancer-TA-MSCs demonstrates excellent predictive accuracy [273]. Similarly, a glioma-TA-MSCs-related gene prognostic index (GA-MSCRGPI) was constructed using eight DEGs, showing a negative correlation with OS and chemoradiotherapy effects. However, patients with high GA-MSCRGPI shows increased sensitivity to immune checkpoint inhibitor (ICI) therapy [274]. A separate study developed a prognostic risk model utilizing MSC abundance. This model predicts poor prognosis and reduced immunotherapy response while indicating prolonged OS in patients receiving anti-angiogenesis treatment [275]. Furthermore, through integration of scRNA-seq data in gastric cancer, a prognostic signature was constructed using seven selected TA-MSC markers. Patients with high-risk scores exhibit worse prognosis but increased sensitivity to antitumor therapy [86]. Apart from the construction of prognosis risk models using MSC-related genes, a recent study integrated scRNA-seq data to generate a distinct stemness-related gene signature involved in the communication between MSCs and CSCs. This gene signature may be exploited to forecast prognosis and immunotherapy response in bladder cancer patients [276].

### Therapeutic intervention

TA-MSCs are crucial in worsening malignant tumor behaviors, making them promising therapeutic targets for tumor management. Current research indicates that therapeutic interventions targeting TA-MSCs can be pursued by either directly inhibiting their formation or eliminating them, which has been succinctly summarized in our previous work [200], as well as by disrupting the interactions between TA-MSCs and other components of the tumor microenvironment, a concept that is well-established. Both approaches are being continuously refined through ongoing advancements in basic and clinical research. These strategies, along with illustrative examples, are discussed in detail below.

The first strategy involves disrupting the recruitment of MSCs to tumor sites. The SDF-1/CXCR4 axis, a central regulator of MSC chemotaxis, can be pharmacologically inhibited by AMD3100, which has been shown to significantly impair MSC homing to tumors and subsequently delay tumor progression [104]. Beyond this pathway, miR-126/miR-126*, which directly targets SDF-1, not only suppresses MSC recruitment but also disrupts the tumor niche, inhibiting lung metastasis in breast cancer models [277]. Another critical target is PDGFRβ, a receptor essential for MSC tropism. A nuclease-resistant RNA aptamer designed against PDGFRβ has demonstrated high specificity in blocking receptor activation, markedly reducing MSC tumor infiltration [278]. Importantly, these chemotactic signals (e.g., SDF-1) often play dual roles: they mediate initial MSC recruitment and facilitate subsequent TA-MSC transformation. Consequently, targeting these pathways offers a two-pronged therapeutic advantage—preventing naïve MSC influx while simultaneously blocking their conversion into TA-MSCs [128]. Given this multifunctional impact, strategies aimed at intercepting MSC recruitment hold exceptional promise for anticancer therapy.

The second strategy seeks to reprogram TA-MSCs to eliminate their tumor-promoting abilities. The activation of YAP signaling is essential for the transition of BM-MSCs into TA-MSCs in gastric cancer, as well as for sustaining their oncogenic phenotype and function.Verteporfin, an inhibitor of YAP signaling, significantly attenuates MSCs’ capacity to enhance lymph node metastasis of cancer cells by disrupting trans-differentiation and reversing the tumor-supportive effects of TA-MSCs [21]. Gastric cancer cell-CM educated BM-MSCs and gastric cancer-TA-MSCs consistently exhibit downregulation of miR-155-5p [130, 279]. Ectopic expression of miR-155-5p inhibits the tumor-promoting effects of TA-MSCs, as its overexpression reverses the phenotypes and functions of gastric cancer-associated TA-MSCs to resemble naïve MSCs [279]. In addition to regulators involved in the transition of naïve MSCs into TA-MSCs that can be intervened to make TA-MSCs “normal”, dysregulated factors in TA-MSCs maintaining their carcinogenic function are another target for intervention. Collagen receptor discoidin domain receptor 2 (DDR2) facilitates metastatic site-TA-MSCs in enhancing breast cancer cells migration, invasion and collagen deposition. Targeted suppression of DDR2 prevents TA-MSCs from promoting the growth and metastasis of breast cancer cells, possibly due to the loss of the mesenchymal phenotype and downregulation of genes related to tumorigenesis in TA-MSCs [20].

The third approach involves directly killing TA-MSCs. Notably, TA-MSCs exhibit heightened vulnerability to ferroptosis compared to normal MSCs, enabling selective targeting using inducers like Erastin and RAS-selective lethal 3 (RSL3). In neuroblastoma models, this approach significantly suppresses tumor growth by preferentially eliminating TA-MSCs [280]. Beyond ferroptosis, tumor-stroma specific targeting strategies show remarkable precision: the FAPα-activated prodrug Z-GP-DAVLBH effectively induces apoptosis in TA-MSCs within triple-negative breast cancer models, substantially reducing metastatic burden in both cell-line derived and patient-derived xenografts [202]. Innovative biological approaches are also emerging, as evidenced by CD8^ +^ T cell-derived EVs that can systemically deplete TA-MSCs to impede tumor progression [281]. Furthermore, engineered oncolytic viruses like Ad5-Ki67/IL-15 demonstrate dual functionality: not only eliminating TA-MSCs but also reversing their immunosuppressive effects on glioma cells through PD-L1 downregulation and angiogenesis inhibition [37]. This multifaceted arsenal of TA-MSC-directed therapies highlights the therapeutic potential of precision stromal targeting in oncology.

The last but not the least approach focuses on disrupting the cross-talk between TA-MSCs and surrounding tumor environment, which includes tumor cells, immune cells, and other components [15, 282]. Research into key communication mediators between TA-MSCs and tumor cells could lead to strategies that block their interaction, offering a strong and lasting therapeutic effect. Gastric cancer cell-derived EVs carrying CD44 induce BM-MSCs transition into TA-MSCs through ERK-PPARγ-CPT1A axis-mediated FAO metabolic reprogramming. This process stimulates MSC secretion of IL-8 and STC1 to promote lymphatic metastasis via enhancing cancer cell migration, invasion and lymphangiogenesis. Inhibiting CD44 expression or FAO activity using etomoxir substantially attenuates lymphatic metastasis by disrupting the interaction between cancer cells and MSCs [133]. Another study identified two interaction loops between MSCs and breast cancer cells. The first loop is composed of CXCL6-CXCR6 and CXCL10-CCR3 for cancer cells recruiting BM-MSCs, while the second loop mediates interaction of TA-MSCs with breast cancer cells. In the second loop, TA-MSCs enhance cancer cells secretion of CSF1 by releasing CCL5, which binds to CCR5 on cancer cells. The cancer cells-derived CSF1 then interacts with the CSF1 receptor on MSCs, facilitating macrophage recruitment and promoting metastasis. Target suppression of CCR5, CCL5 or CSF1 or both by knockdown of HIF significantly suppresses primary tumor growth and metastasis [129]. Besides, lipid-lowering drugs like statins and nanoparticles are being studied for their potential to interfere with the interaction between MSCs and tumor cells in tumor therapy [46, 283]. As to immune cells, TA-MSCs suppress their anti-tumor immunity within both the innate and adaptive immune systems [33, 47, 203, 216]. Targeting the factors that mediate this suppression could enhance immunotherapy [203, 208, 209, 216]. For instance, ovarian tumor-TA-MSCs recruit immunosuppressive monocytes and induce the polarization of M2-type macrophages (CCR2^+^Tgfbi^+^ myeloid cells) to promote the exclusion of CD8^+^ T cells and impairment of NK cell activity, thus resisting anti-PD-L1 immunotherapy. Targeting hedgehog signaling in TA-MSCs reverses their immune-suppressive function, thereby improving responses to ICI therapy [203]. TA-MSCs secret CXCL5, NO, and GM-CSF, which promote the expansion of MDSCs to suppress T cell activity, thereby exerting immunosuppressive functions. Targeted inhibition of these secreted factors alters the immunosuppressive effects of TA-MSCs, thus increasing sensitivity to anti-PD-L1 therapy [205]. Immune cells, including neutrophils and CD4^+^ T cells, also play a role in the tumor-promoting function of TA-MSCs [204, 284]. Th2 cells is essential for mouse MSCs in the pre-metastatic niche to express complement 3 (C3), thereby acquiring the ability to recruit neutrophils, promote their aggregation, and induce the formation of NETs. This cascade of events, in turn, facilitates the lung metastasis of breast cancer cells [27]. Similarly, targeted disruption of the interaction between immune cells and TA-MSCs presents another promising strategy for tumor treatment. Regarding other components in the TME, TA-MSCs promote angiogenesis by directly or indirectly enhancing angiogenic sprouting and tube formation of endothelial cells. The involved factors such as STC1, VEGF and HIF-1α are expected to be intervened to prevent tumor growth and metastasis [133, 196, 204]. Furthermore, the selective inhibition of DDR2 effectively hinders breast cancer metastasis-associated TA-MSCs from depositing collagen type I, thereby reducing their capacity to facilitate breast cancer progression [20]. While the pivotal roles of the ECM in tumor progression have long been appreciated [285, 286], the impact of TA-MSCs on ECM remains understudied. Future research should aim to clarify the mechanisms in this field to discover new therapeutic targets.

## Perspectives and conclusions

MSCs are ideal for cell-based therapies due to their low immunogenicity and ability to differentiate into various stromal cell types. Over recent decades, numerous clinical trials have employed naïve MSCs for the treatment of diverse pathologies, highlighting the critical importance and urgency of MSC applications in regenerative medicine [287]. However, during this same period, conflicting findings have emerged regarding the role of naïve MSCs in tumorigenesis, alongside an increasing identification of TA-MSCs. These developments have prompted researchers to re-evaluate the safety of MSC-based therapies, thereby opening new avenues for exploring the mechanisms underlying tumor initiation and progression from the unique perspective of MSCs. Current research primarily seeks to address two fundamental questions: the processes involved in the generation of TA-MSCs and the mechanisms by which TA-MSCs exacerbate tumor behavior. The findings from these studies have indeed provided valuable insights into the heterogeneity, functions, regulatory mechanisms, and clinical relevance of TA-MSCs. Despite a significant body of related research, the proportion of TA-MSCs within tumor tissues has received limited attention. Although existing markers facilitate the determination of their proportion through flow cytometry or single-cell sequencing analyses, the reliability of these findings remains questionable. Our knowledge of TA-MSCs’ characteristics and biological functions mainly stems from research involving the isolated TA-MSCs and tumor cell-educated MSCs. However, the extent to which various culture conditions and artificial factors in vitro accurately reflect the in vivo functions of TA-MSCs is a matter of debate. Therefore, it is essential to explore these and other nuanced aspects of research in greater depth to achieve a more comprehensive understanding of TA-MSCs. The following critical issues must be addressed before TA-MSCs can be effectively employed in clinical applications:

(I) Lack of specific biomarkers for identifying and tracking TA-MSCs. Although the ISCT criteria are employed to characterize TA-MSCs, these markers are not exclusive to them. At present, the heterogeneity of TA-MSCs poses a significant challenge in the identification of specific biomarkers, representing the primary obstacle in TA-MSC research. Developing systems for cell lineage tracing and genetic manipulation using specific biomarkers is crucial for studying MSCs’ role in tumor development. These biomarkers enable the isolation, quantification, and characterization of pure TA-MSCs, as well as targeted interventions to mitigate adverse effects.
(II) Ongoing debate between TA-MSCs and CAFs: A comprehensive analysis of recent studies highlights notable similarities in the methodologies employed to isolate TA-MSCs and CAFs from tumor tissues. Currently, the terminology used to describe these cells may largely reflect researchers’ preferences. Although each study has undertaken efforts to identify the cells, the characterization techniques, including cell morphology and marker gene expression, lack specificity. The potential for multiple differentiation could serve as a crucial criterion for distinguishing TA-MSCs from CAFs. However, this assay is predominantly performed in studies focusing on TA-MSCs, rather than those examining CAFs, thereby leaving unresolved questions about whether CAFs possess multi-lineage differentiation potential. Intriguingly, a study has shown that primary human neuroblastoma tumors-derived CAFs share tri-lineage differential capacity with MSCs [100], highlighting a notable similarity between TA-MSCs and CAFs. Pancreatic cancer TA-MSCs, isolated from CAFs, exhibit notable differentiation potential and an hightened capacity to facilitate tumor progression compared to other CAF subpopulations without TA-MSCs. This suggests that TA-MSCs may constitute a distinct and essential subpopulation of CAFs involved in tumor progression [78]. Conversely, in a scRNA-seq data analysis, Qi* et al.* classified CAFs as a subgroup of MSCs in colon cancer [288]. Notably, MSCs in this study were identified using maker genes *COL1A1* and *COL3A1*, which are commonly employed for annotating fibroblast populations in scRNA-seq data from other research groups. These findings suggest that TA-MSCs and CAFs obtained from tumor tissue are essentially the same cell population and may be more appropriately described as “certain specific markers positive or negative stromal cells”.
(III) Ununified experimental and culture conditions of MSCs: Discrepancies observed in MSC functions have been attributed to variations in contact culture systems and models [45, 47, 171], utilization of MSCs at different passages and confluences [70, 289], as well as differences in the timing of exogenous MSC administration [290, 291]. Hence, it is imperative to provide comprehensive experimental details in each study to establish a standardized framework for MSC conditions. This approach ensures the comparability and reproducibility of MSC research outcomes across different laboratories. Additionally, the current findings primarily stem from various artificial models involving exogenous MSC administration and warrant critical evaluation. Future research efforts should prioritize investigating endogenous MSCs to yield more objective and convincing results.

In conclusion, TA-MSCs have been extensively validated as components of the TME, exhibiting heterogeneity in terms of cellular origin, phenotype, plasticity, and function. Despite this variability, they consistently contribute to tumor initiation, progression, and metastasis. TA-MSCs hold significant promise for applications in tumor diagnosis, prognosis prediction, and therapy. Addressing key challenges is essential, such as identifying specific markers for TA-MSCs, clarifying their relationship with CAFs, and the standardizing experimental conditions for TA-MSCs. These efforts are vital for a comprehending the heterogeneity and roles of TA-MSCs, and are fundamental for developing innovative diagnostic and therapeutic strategies involving TA-MSCs.

## Data Availability

No datasets were generated or analysed during the current study.

## Abbreviations

5-FU: Fluorouracil
ANXA2: Annexin A2
BMP: Bone morphogenetic protein
C3: Complement 3
CAF: Cancer-associated fibroblast
CFU-F: Colony-forming unit fibroblasts
circRNAs: Circular RNAs
CSCs: Cancer stem cells
CTLs: Cytotoxic lymphocytes
DCs: Dendritic cells
DDR2: Discoidin domain receptor 2
DEGs: Differentially expressed genes
DEN: Diethylnitrosamine
DIM: 3,3’-diindolylmethane
ECM: Extracellular matrix
EGF: Epidermal growth factor
ER^+^: Estrogen receptor-positive
EVs: Extracellular vesicles
FAO: Fatty acid oxidation
GA-MSCRGPI: Glioma-TA-MSCs-related gene prognostic index
GM-CSF: Granulocyte-macrophage colony-stimulating factor
GSC: Gastric carcinoma
HA: Hyaluronic acid
HCC: Hepatocellular carcinoma
HGF: Hepatocyte growth factor
HG-SOCs: High-grade serous ovarian cancers
HLA-I: HLA class I molecules
HNSCC: Head and neck squamous cell carcinoma
H. _pylori_: Helicobacter pylori
ICI: Immune checkpoint inhibitor
IDO: Indoleamine 2,3-dioxygenase
iNOS: Inducible nitric oxide synthase
ISCT: International Society for Cellular Therapy
LC: Lung carcinoma
LIF: Leukemia inhibitory factor
lncRNA: Long non-coding RNA
LOX: Lysyl oxidase
MARCKS: Myristoylated alanine-rich C-kinase substrate
MDSCs: Myeloid-derived suppressor cells
MET: Mesenchymal-to-epithelial transition
miRNAs: MicroRNAs
MSCs: Mesenchymal stromal/stem cells
NETs: Neutrophil extracellular traps
NO: Endogenous nitric oxide
NOS: Nitric oxide synthase
NPPB: Natriuretic peptide B
NPRA: Natriuretic peptide receptor A
NTA-MSCs: Non-tumor tissues derived MSCs
OS: Overall survival
OXPHOS: Oxidative phosphorylation
PBMCs: Peripheral blood mononuclear cells
PDGF-B: Platelet-derived growth factor-B
PFS: Progression-free survival
PGE2: Prostaglandin E2
RSL3: RAS-selective lethal 3
scRNA-seq: Single-cell RNA sequencing
SIRT1: Sirtuin 1
STC1: Stanniocalcin-1
TA-MSCs: Tumor-associated MSCs
THBS2: Thrombospondin-2
TME: Tumor microenvironment
TNFα: Tumor necrosis factor α
tNRG1: Transmembrane neuregulin 1
VM: Vascular mimicry
XIAP: X-linked inhibitor of apoptosis protein
